# Ketamine persists within neuronal compartments long after systemic clearance

**DOI:** 10.21203/rs.3.rs-10234621/v1

**Published:** 2026-07-22

**Authors:** Joseph Cichon, Kallol Bera, Zachary Blumenfeld, Elaine Lin, Meah Ahmed, Eve Fine, Md. Nadim Hossain, Andrey Andreev, Altyn Rymbek, Peter Fenton, Helena Ambrosino, Sara Zimmerman, Jacqueline Morris, Aalok Varma, Bruce Cohen, Dennis Dougherty, David Prober, Henry Lester, Loren Looger

**Affiliations:** University of Pennsylvania; University of California, San Diego; University of Southern California, Los Angeles; California Institute of Technology; University of Pennsylvania; California Institute of Technology; University of California, San Diego; California Institute of Technology; California Institute of Technology; University of California, San Diego; California Institute of Technology; University of California, San Diego; University of California, San Diego; University of California, San Diego; California Institute of Technology; California Institute of Technology; California Institute of Technology; California Institute of Technology; University of California, San Diego

## Abstract

Ketamine produces rapid and sustained antidepressant effects after a single dose. Plasma membrane NMDA receptors are the presumptive primary molecular targets, yet how ketamine distributes, localizes, and engages neurons across cellular and subcellular compartments remains largely unknown. Ketamine, a lipophilic weak base, is thought to accumulate within acidic intracellular compartments, suggesting potential unrecognized sites of action. We developed a toolbox of genetically encoded intensity-based ketamine-sensing fluorescent reporters (iKetSnFRs) that specifically and robustly report R- or S-ketamine with cellular and subcellular resolution *in vitro* and *in vivo*, with excellent sensitivity and fluorescence change. iKetSnFR enables rapid detection of ketamine within seconds in the mouse prefrontal and visual cortex after intranasal or intraperitoneal dosing. We subsequently engineered compartment-targeted iKetSnFR variants, and *in vivo* imaging revealed that ketamine remains intracellularly and subcellularly engaged in neurons well after it is no longer detectable in circulation by LC-MS, at the plasma membrane of cortical neurons by iKetSnFR, or by electroencephalography. Furthermore, iKetSnFRs targeted across nine compartments reveal organelle-specific engagement of ketamine. This persistent intracellular engagement coincides with the prolonged timeframe of behavioral effects. Together, these findings link a single ketamine exposure to persistent intracellular drug dynamics and begin to provide a mechanistic basis for its rapid and enduring cellular and behavioral effects.

## INTRODUCTION

Ketamine, originally described as a dissociative anesthetic, produces rapid and sustained antidepressant effects at subhypnotic doses, establishing it as the founding rapid-acting antidepressant (RAAD)^1–6^. How this rapid yet long-lasting efficacy is mediated is poorly understood, which hinders the development of next-generation RAADs. Despite promising results in clinical practice for treatment-resistant depression, the effect sites and molecular mechanisms of ketamine action remain incompletely understood. Antagonism of N-methyl-D-aspartate (NMDA) and other glutamate receptors^7–10^ is clearly important, with additional candidate molecular targets including nicotinic acetylcholine receptors, dopamine D2 receptors, HCN1 channels, and serotonin receptors^11,12^, among others (with ketamine assumed to act at the plasma membrane for all targets). Subhypnotic ketamine activates diverse downstream signaling pathways, including mTOR, EEF2, GSK-3, CaMKII, BDNF–TRKB, *etc*. in prefrontal, hippocampal, and habenular networks^13–19^. Additionally, R- and S-ketamine are increasingly recognized as distinct RAADs: S-ketamine produces more transient, NMDA receptor–dependent antidepressant effects accompanied by greater dissociation, whereas R-ketamine elicits more sustained antidepressant responses with fewer psychotomimetic effects, likely through longer-lasting plasticity mechanisms^20–23^.

While most mechanistic studies have focused on cell-surface receptor engagement and downstream signaling, ketamine’s chemical properties suggest that it may also accumulate within intracellular compartments and organelles, where it could persistently influence additional pathways. Because ketamine is a weak base with a pK_a_ of 7.3, roughly 50% of ketamine molecules exist in the deprotonated, membrane-permeable form at physiological pH, enabling efficient passive diffusion across lipid bilayers^24^. Ketamine’s moderate lipophilicity (logP ~ 3.35) and low hydrogen-bonding capacity—one donor and two acceptors—further support broad membrane permeability. As early as 1974, de Duve proposed that weak bases can accumulate more than 100-fold within acidic organelles such as lysosomes and synaptic vesicles^25,26^. Subsequent studies validated this concept with nicotine, demonstrating that lysosomal trapping can profoundly shape pharmacological actions^27^. Although acidic vesicles (which comprise ~ 1% of the cell on average) and other organelles are small in volume, they can sequester amounts of weakly basic drugs, comparable to that in the entire cytoplasm^24^. As one consequence, lipophilic weak bases such as antipsychotics accumulate within synaptic vesicles (pH ~ 5.5) and can be released in an activity-dependent manner, modulating postsynaptic signaling^28,29^ - a mechanism critical to their function. It is possible that ketamine could also accumulate in other hydrophobic compartments.

Ketamine displays rapid absorption and distribution in both humans and rodents. In humans, intravenous ketamine yields immediate central nervous system (CNS) penetration and biphasic clearance, with a distribution half-life of ~ 10–15 min and a terminal elimination half-life of 2–3 h. The drug undergoes extensive metabolism in the liver and elsewhere, producing norketamine (NK), hydroxynorketamine (HNK), and dehydronorketamine (DHNK) metabolites with substantially longer half-lives^20,30,31^, leading to sustained plasma and cerebrospinal fluid (CSF) levels^32^. In mice, after intraperitoneal (i.p.) dosing, peak plasma and brain ketamine concentrations are reached within 5–10 min, and the terminal half-life is only 30–60 min^33^. Rodents also show a higher fractional conversion to HNK/DHNK, presumably due to species-specific cytochrome P450 activity^34,35^. These differences compress the window of ketamine-receptor interactions in mice, whereas humans experience more prolonged exposure and higher metabolite accumulation. Most ketamine pharmacokinetic (PK) estimates come from blood plasma, not the brain^36,37^. Direct brain^36–38^ and intracellular pharmacokinetics remain essentially unknown, underscoring the need for *in vivo* brain-level and subcellular readouts.

We propose that ketamine undergoes dramatic neuronal compartmental trapping, with significant accumulation in subcellular compartments serving as previously unrecognized sites of action. To test this hypothesis, we developed novel genetically encoded fluorescent ketamine biosensors, iRKetSnFR2.0 and iSKetSnFR3.0, capable of detecting individual ketamine enantiomers at the level of individual neurons and across distinct subcellular compartments. These tools reveal that ketamine’s presence is spatially diverse and more prolonged than blood plasma PK measurements suggest, reframing fundamental assumptions about how ketamine acts within cells and the brain. We propose that ketamine’s near-instantaneous uptake into neurons and extended intracellular engagement *in vivo* could underlie its functions as a rapid-onset and long-lasting antidepressant, respectively.

## RESULTS

### Ketamine biosensor design and screening

All sensors described here descend from the original iNicSnFR genetically encoded nicotine sensor construct^39^, which consists of a extensively mutated acetylcholine-binding periplasmic binding protein (PBP) fused to circularly permuted GFP (cpGFP). Except for organelle targeting sequences, purification handles, and antibody tags, all variants within the family are developed *via* point substitutions, preserving the length (522 residues). All variants we discuss have a Hill coefficient near 1. Therefore, we use the S-slope [defined as (ΔF_max_/F_0_)/EC_50_]^40^ as the best measure of linear sensor performance at concentrations less than the *EC*_50_.

**Figure 1:**
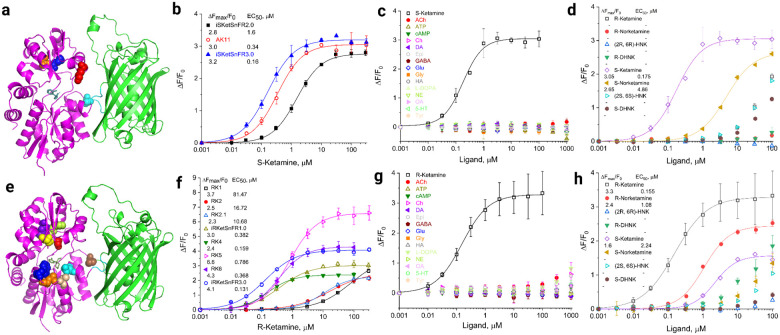
Structure-guided engineering and characterization of ketamine-sensitive fluorescent sensors. **(a)** AlphaFold3 docking model of iSKetSnFR3.0 in complex with S-ketamine, shown in ribbon representation. Mutated residues are shown as spheres. The periplasmic binding protein (PBP) domain is colored magenta, and the circularly permuted green fluorescent protein (cpGFP) domain is shown in green. “Bottom lobe” of the PBP is defined as the location of the N- and C-termini. Residues are color-coded as follows: 359M (blue), 360A (yellow). **(b)** Characterization of S-ketamine sensors. Dose–response curves of iSKetSnFR and successively more sensitive variants (iSKetSnFR2.0, AK11, iSKetSnFR3.0) to S-ketamine. Fluorescence responses are plotted as ΔF/F_0_ versus ligand concentration (μM). Inset table summarizes ΔF_max_/F_0_, and EC_50_. Progressive engineering improved both sensitivity (lower EC_50_) and total fluorescence change. **(c)** Ligand selectivity of the optimized S-ketamine sensor (iSKetSnFR3.0). Responses to a panel of neurotransmitters and related small molecules are shown relative to S-ketamine (black). The sensor exhibits high specificity with negligible off-target responses. Dose-response analysis (pH 7.0, 3× PBS) yields ΔF_max_/F_0_ = 3.2 ± 0.3, EC_50_ = 160 ± 20 nM, S-slope = 20 μM^−1^, and Hill coefficient n_H_ = 1.18 ± 0.07. **(d)** Cross-sensitivity of iSKetSnFR3.0 with ketamine enantiomers and their metabolites. Dose–response curves for S-ketamine, R-ketamine, and ketamine metabolites [R-norketamine, S-norketamine, (2R, 6R)-HNK, (2S, 6S)-HNK, R-DHNK, S-DHNK] are shown. Inset parameters show ΔF_max_/F_0_, and EC_50_. **(e)** AlphaFold3 docking model of iRKetSnFR2.0 bound to R-ketamine. Mutated residues are shown as spheres. Domain coloring is as in **(a)**. Residues are color-coded as follows: 10L (orange), 12W (blue), 43V (wheat), 68Q (cyan), 359F (green-cyan), 360M (red), and 435V (yellow). **(f)** Characterization of R-ketamine sensors. Dose–response curves of iRKetSnFR variants (RK1, RK2, RK2.1, RK2.2, iRKetSnFR1.0, RK4, RK5, RK6, and iRKetSnFR2.0) to R-ketamine. Fluorescence responses (ΔF/F_0_) are plotted against ligand concentration. Inset summarizes ΔF_max_/F_0_, and EC_50_, demonstrating progressive improvements in sensitivity and signal amplitude. **(g)** Ligand selectivity of iRKetSnFR2.0. Fluorescence responses to neurotransmitters and related small molecules are shown relative to R-ketamine (black) and show strong selectivity for R-ketamine. Dose–response analysis (pH 7.0, 3× PBS) yields ΔF_max_/F_0_ = 4.1 ± 0.3, EC_50_ = 130 ± 20 nM, and n_H_ = 0.87 ± 0.07. **(h)** Cross-sensitivity of iRKetSnFR2.0 with ketamine enantiomers and their metabolites. Dose–response curves of iRKetSnFR2.0 vs. ketamine enantiomers and ketamine metabolites [R-norketamine, S-norketamine, (2R, 6R)-HNK, (2S, 6S)-HNK, R-DHNK, S-DHNK] reveal differential sensitivity, highlighting stereoselective detection of R-ketamine. Data are presented as mean ± s.d. from 3 independent experiments (biological replicates). All curves were fitted using the Hill equation.

Our previously developed first-generation ketamine biosensor, iSKetSnFR2.0, is highly sensitive to S-ketamine, exhibiting an S-slope of 1.87 μM^−140^. To overcome the many hurdles to photon collection presented by *in vivo* and subcellular imaging and to extend sensor utility to low drug concentrations, we first aimed to engineer next-generation variants with markedly enhanced sensitivity and fluorescence increase. Accordingly, we generated and characterized ~ 50,000 variants of each iKetSnFR construct through iterative rounds of site-saturated mutagenesis (SSM) at various protein sites (**Extended Data** Fig. 1)^41,42^. Mutations at positions 359 and 360 in the upper lobe of the binding pocket of iSKetSnFR2.0 substantially increased sensitivity. Specifically, T360A alone yielded an improved variant, AK11, with an S-slope of 8.9 μM^−1^, and the addition of A359M further optimized the sensor, generating iSKetSnFR3.0 (Fig. 1a–b), with an S-slope of 20 μM^−1^—representing a > 10x increase in sensitivity for S-ketamine detection over iSKetSnFR2.0 (Fig. 1b).

Having made a robust sensor for S-ketamine, we next sought to make one appropriate for detecting its enantiomer, R-ketamine. We began with the AK1 scaffold, which exhibited only a weak response to S-ketamine (ΔF/F_0_ ~ 0.12 at 1 μM)—but no response to R-ketamine^40^. SSM at His68 revealed that a single substitution to Gln conferred a detectable but small response to R-ketamine (ΔF/F_0_ ~ 0.11 at 1 μM), yielding the prototype RK1. Iterative SSM at residues adjacent to the ligand-binding pocket (E43V, I10L, A435V, and F12W, in that order) progressively reshaped the binding pocket and produced iRKetSnFR1.0, which showed a marked increase in sensitivity, with an S-slope of 7.9 μM^−1^ and a ΔF/F_0_ of ~ 7.9 at 1 μM R-ketamine. Subsequent mutations of linker-2 and additional pocket residues (324, 359, and 360) further enhanced performance, culminating in iRKetSnFR2.0, with an S-slope of 31.0 μM^−1^ (Fig. 1e–f; **Extended Data** Fig. 1).

### Biophysical characterization of iRKetSnFR2.0 and iSKetSnFR3.0

We first systematically evaluated the performance of iSKetSnFR3.0 and iRKetSnFR2.0 in terms of affinity, rise and decay kinetics, fluorescence change, and binding selectivity across a comprehensive panel of neurotransmitters (Fig. 1c, g) and metabolites (Fig. 1d, h). Dose-response titrations revealed that both sensors exhibited robust, concentration-dependent fluorescence changes [nanomolar (nM)-to-micromolar (μM) sensitivity] in response to their cognate ketamine enantiomer, while remaining unresponsive to a diverse panel of neurotransmitters, including glutamate, GABA, dopamine, serotonin, acetylcholine, and norepinephrine (Fig. 1c, g). iSKetSnFR3.0 exhibited high sensitivity for S-ketamine (*EC*_50_ of 175 ± 20 nM), whereas S-norketamine displayed ~ 28-fold lower affinity (*EC*_50_ = 4.86 μM), and HNK and DHNK metabolites produced only weak responses at high concentrations (Fig. 1d). Similarly, iRKetSnFR2.0 preferentially detected R-ketamine (*EC*_50_ = 0.155 μM), with reduced sensitivity to R-norketamine (*EC*_50_ = 1.08 μM) and minimal activation by HNK and DHNK metabolites (Fig. 1h). This molecular specificity ensures that fluorescence signals reflect ketamine enantiomers at pharmacologically relevant concentrations.

**Figure 2:**
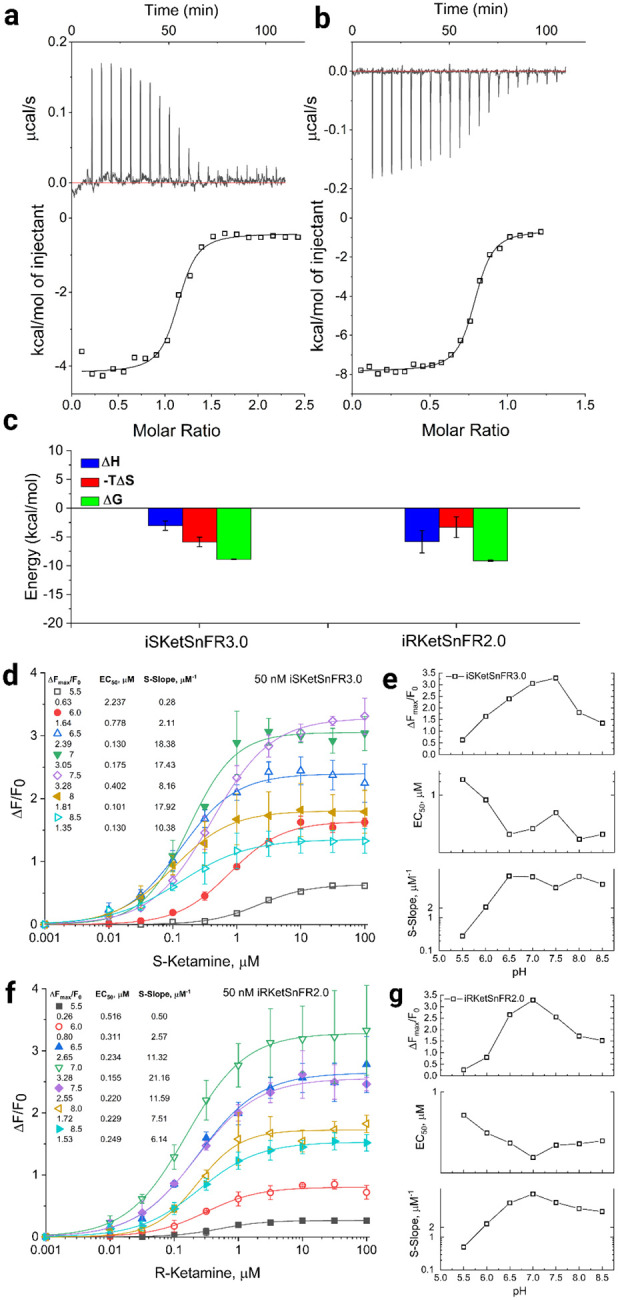
Thermodynamic and photophysical characterization of iRKetSnFR2.0 and iSKetSnFR3.0. **(a)** Representative isothermal titration calorimetry (ITC) thermogram (top) and corresponding binding isotherm (bottom) for S-ketamine binding to iSKetSnFR3.0. Raw heat release per injection (μcal/s) is plotted versus time, and integrated heats are plotted against molar ratio and fit to a single-site binding model to determine thermodynamic parameters. The integrated heats of injection yield a dissociation constant (K_d_ = 296 ± 27 nM) that is consistent with the fluorescence-derived EC_50_ (~180 nM), confirming high-affinity ligand recognition. **(b)** ITC thermogram (top) and binding isotherm (bottom) for R-ketamine binding to iRKetSnFR2.0. Differences in binding enthalpy and isotherm shape indicate distinct energetic contributions to ligand-sensor binding. The isotherm reveals a high-affinity interaction (K_d_ = 120 ± 5 nM) in agreement with the fluorescence EC_50_ (~150 nM). **(c)** Thermodynamic parameter decomposition of ligand binding for iSKetSnFR3.0 and iRKetSnFR2.0. Bar plots show enthalpy change (ΔH, blue), entropic contribution (−TΔS, red), and free energy change (ΔG, green). Both sensors exhibit favorable binding free energy, with differing balances of enthalpic and entropic contributions, suggesting distinct ligand recognition and conformational changes. **(d)** Dose–response curves of purified iS-KetSnFR3.0 [50 nM] to increasing concentrations of S-ketamine [0.001–100 μM] at varying pH conditions (5.5–8.5) [λ_ex_ = 480 nm; λ_em_ = 535 nm]. Fluorescence responses are plotted as ΔF/F_0_ versus ligand concentration (μM). The sensor exhibits strong pH dependence, with maximal dynamic range and affinity observed near neutral pH. **(g)** Summary of pH dependence of iSKetSnFR3.0 properties. Data were fit to a Hill equation to extract apparent EC_50_, maximal fluorescence change (ΔFmax/F_0_), and S-slope (μM^−1^). **(f)** Dose–response curves of purified iRKetSnFR2.0 [50 nM] to increasing concentrations of R-ketamine [0.001–100 μM] across a range of pH conditions (5.5–8.5) [λ_ex_ = 480 nm; λ_em_ = 535 nm]. **(e)** Summary of pH dependence for iRKetSnFR2.0. These data highlight pH-dependent modulation of both sensitivity and dynamic range.

We next defined the thermodynamic basis of ligand binding with isothermal titration calorimetry (ITC) (Fig. 2a–c). The dissociation constant (K_d_) for iSKetSnFR3.0 was 300 ± 27 nM, closely matching its *EC*_50_ of 175 ± 20 nM from fluorescence titration, indicating that fluorescence accurately reflects drug levels rather than other confounding factors (Fig. 2a). Similarly, iRKetSnFR2.0 exhibited a K_d_ of 120 ± 5 nM by ITC and a fluorescence *EC*_50_ of 155 ± 10 nM (Fig. 2b); thus, the fluorescence signals of both biosensors directly reflect the concentration of the corresponding ketamine enantiomer. Thermodynamic analysis revealed distinct energetically favorable binding modes (Fig. 2c). S-ketamine binding to iSKetSnFR3.0 and R-ketamine binding to iRKetSnFR2.0 were both exothermic (ΔH < 0); however, S-ketamine/iSKetSnFR3.0 had a favorable entropic contribution (ΔS > 0), while R-ketamine binding to iRKetSnFR2.0 had ΔS < 0 and was dominated by enthalpic stabilization. Of note, SnFRs that we have previously developed for fluoxetine and escitalopram^43^ (from the same iNicSnFR parent) also show major differences in ΔS, perhaps because the various drug binding sites in this SnFR family trap or release differing numbers of water molecules.

To assess pH sensitivity and performance under conditions relevant to blood, CSF, cells, and organelles, we determined dose-response titrations using 50 nM purified sensor and 0–100 μM S-ketamine across pH 5.0–8.5 (Fig. 2d–g, **Extended Data** Fig. 2). iSKetSnFR3.0 exhibited a maximal S-slope of ~ 18 μM^−1^ at pH 6.5, with sufficiently robust responses at pH 6.0 (S-slope ~ 2 μM^−1^, ΔF_max_/F_0_ = 1.64, *EC*_50_ = 780 nM), suitable for imaging within the mildly acidic Golgi (pH 6.0–6.5). Consistent with the “candle snuffer” mechanism observed from atomic-scale structures of the iNicSnFR family^39^, fluorescence amplitude decreased sharply under more acidic conditions (iSKetSnFR3.0 ΔF_max_/F_0_ ~ 0.6 at pH 5.5, ~ 5-fold lower than at pH 7.0), rendering the current sensor unsuitable for endolysosomal environments. iSKetSnFR3.0 performed optimally between pH 6.5–8.0, where ΔF_max_/F_0_ increased with pH until 7.5, before declining due to elevated baseline fluorescence (*F*_0_). Similarly, iRKetSnFR2.0 displayed a maximum S-slope of ~ 21 μM^−1^ at pH 7.0 and retained modest responsiveness at pH 6.0 (S-slope ~ 2.6 μM^−1^, ΔF_max_/F_0_ = 0.9, *EC*_50_ = 310 nM). Acidic pH strongly attenuated ΔF_max_/F_0_ (~ 0.25 at pH 5.5, ~ 13-fold lower than at pH 7.0), indicating that the sensor performs best in neutral to mildly alkaline environments (Fig. 2e, g). Collectively, these results define the optimal operating range, selectivity, and binding thermodynamics of the iKetSnFR family, establishing a quantitative framework for interpreting sensor response magnitude and kinetics *in vivo*. Importantly, the sensors have appropriate binding affinity for physiological ketamine concentrations, excellent ligand-binding selectivity for the appropriate ketamine enantiomer over the other enantiomer and endogenous molecules, large ligand-dependent fluorescence increases, and sufficient pH resistance to allow imaging in the cytoplasm and the subcellular compartments most relevant to ketamine trafficking and action.

### In vitro iKetSnFR imaging reveals ketamine’s rapid membrane permeation and intracellular access

**Figure 3:**
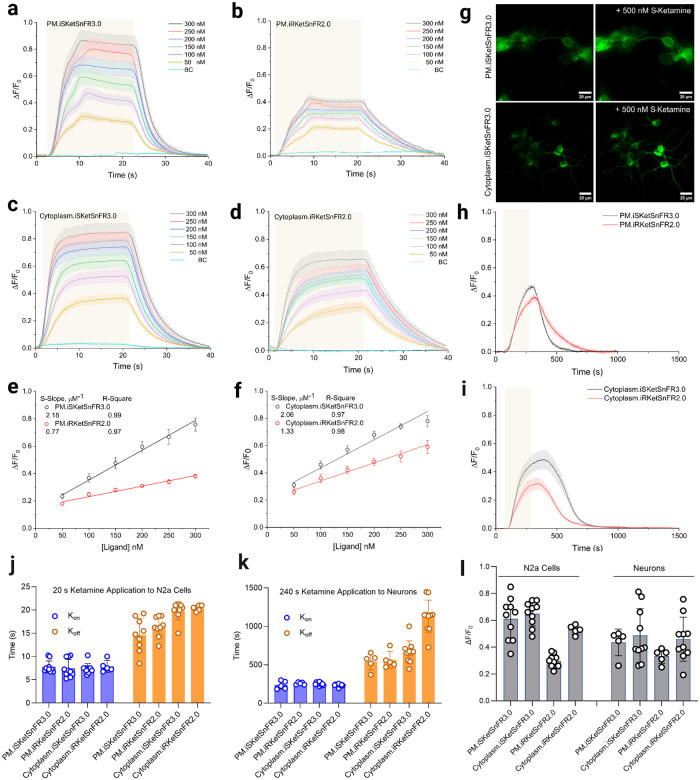
*In vitro* dose–response relationships of R- and S-ketamine using iKetSnFRs. **(a, b)** Representative fluorescence responses (ΔF/F_0_) of plasma membrane (PM)-targeted iSKetSnFR3.0 (n = 10) (a) and iRKetSnFR2.0 (n = 10) (b) expressed in Neuro2a (N2a) cells during a 20 s application of increasing ketamine concentrations (50–300 nM; shaded region). Buffer control (BC) traces are shown in cyan. **(c, d)** Representative fluorescence responses of cytoplasm-targeted iSKetSnFR3.0 (n = 10) (c) and iRKetSnFR2.0 (n =10) (d) under identical ketamine application (50–300 nM, 20 s) conditions. **(e)** Concentration–response relationships for PM-targeted sensors. Linear regression analysis demonstrates greater sensitivity of PM.iSKetSnFR3.0 (black; slope = 2.18 μM^−1^, R^2^ = 0.99) compared with PM.iRKetSnFR2.0 (red; slope = 0.77 μM^−1^, R^2^ = 0.97). Data are mean ± SEM. **(f)** Concentration–response relationships for cytoplasm-targeted sensors. Cytoplasm.iSKetSnFR3.0 (black; slope = 2.06 μM^−1^, R^2^ = 0.97) exhibits higher sensitivity than cytoplasm.iRKetSnFR2.0 (red; slope = 1.33 μM^−1^, R^2^ = 0.98). (**g**) Subcellular localization of plasma membrane (PM)-, and cytoplasm-targeted iSKetSnFR3.0 biosensors in primary cortical neurons before and after S-ketamine exposure (500 nM). **(h, i)** Representative fluorescence recordings from primary cortical neurons expressing PM-targeted (**h**) or cytoplasm-targeted (h) iKetSnFRs during a 240-s application of 500 nM ketamine enantiomer followed by washout. Insets show representative fluorescence images of neurons expressing each sensor. Scale bars, 20 μm. PM-targeted sensors exhibited rapid fluorescence increases and washout decay time constants of 542 ± 108 s for S-ketamine and 564 ± 109 s for R-ketamine. Cytoplasm-targeted sensors displayed comparable peak amplitudes but slower clearance kinetics, with decay time constants of 665 ± 147 s for S-ketamine and 1130 ± 203 s for R-ketamine. **(j)** Quantification of K_on_ (blue) and K_off_ (orange) time constants in N2a cells. K_on_ is defined as the time constant describing the increase in fluorescence from the baseline signal at the onset of ketamine application to the steady-state fluorescence plateau achieved during ketamine application. K_off_ is defined as the time constant describing the decay in fluorescence from the steady-state plateau back to baseline immediately after termination of ketamine bath application. Individual circles represent independent cells (n = 5–10). **(k)** Quantification of K_on_ and K_off_ kinetics in primary cortical neurons during a 240-s ketamine application. Individual circles represent independent neurons (n = 5–10). **(l)** Quantification of peak responses (ΔF_max_/F_0_) for PM- and cytoplasm-targeted iSKetSnFR3.0 and iRKetSnFR2.0 in N2a cells at 200 nM ketamine (left) and in primary cortical neurons at 500 nM ketamine (right). Individual circles represent independent measurements; bars or solid lines indicate mean ± s.e.m.

To validate iKetSnFR performance under controlled cellular conditions prior to *in vivo* imaging, we first assessed iSKetSnFR3.0 and iRKetSnFR2.0 responses to ketamine in cultured Neuro2a (mouse) cells at the plasma membrane (PM), thus revealing the presence of the drug at the outside of the cell surface [**Table 1; Table 2**]. Cells expressing sensors were imaged using widefield epifluorescence microscopy with rapid perfusion to enable control of extracellular drug concentrations. Ligand concentrations (≤ 300 nM) were selected to span the linear range of the sensors based on purified-protein EC_50_ values. As expected, PM-displayed sensors exhibited rapid, concentration-dependent increases in fluorescence (K_on_ = 7.7 ± 1.3; 7.5 ± 1.9 s) in response to both 300 nM S- and R-ketamine, followed by rapid decay upon washout (K_off_ = 14.6 ± 3.6; 16.4 ± 2.2 s) (Fig. 3a, b, h), consistent with fast equilibration of extracellular ketamine. K_on_ is defined as the time constant (s) describing the increase in fluorescence from the baseline signal at the onset of ketamine application to the steady-state fluorescence plateau achieved during continuous ketamine exposure. K_off_ is defined as the time constant (s) describing the decay in fluorescence from the steady-state plateau back to baseline immediately after termination of ketamine perfusion. At 300 nM, both sensors produced robust ΔF/F_0_ signals across cell types: in Neuro2a cells, S-ketamine, 0.65 ± 0.04 and R-ketamine, 0.85 ± 0.08 (Fig. 3a–d). PM responses in SH-SY5Y cells also produced comparable (ΔF_max_/F_0_) responses like neuro 2a cells: S-ketamine, 0.58 ± 0.04 and R-ketamine, 0.26 ± 0.02 (**Extended Data** Fig. 3).

We next restricted iKetSnFR localization to the cytoplasm (and out of the nucleus, to which cytoplasmic proteins can readily exchange) by incorporating a nuclear exclusion signal (NES) at the N-terminus of the construct [**Table 1; Table 2**]. This strategy is similar to what we have previously done with red calcium indicators like RCaMP and RGECO^44^; the green calcium indicator GCaMP has a cryptic internal NES and thus is naturally nucleus-excluded^45^. Here, both cytoplasmic iKetSnFRs exhibited robust, concentration-dependent fluorescence increases, demonstrating rapid cell uptake following drug application. Signal rises occurred within seconds of perfusion (7.4 ± 1.1; 7.9 ± 1.2 s) and decayed upon washout (19.8 ± 1.9; 20.4 ± 6.4 s), consistent with efficient membrane permeation and intracellular localization of S-ketamine and R-ketamine, respectively. Response amplitudes and kinetics were similar across cell types (rise time constant of ~ 7 ± 1 s and a washout decay time constant of ~ 20 ± 5 s at 200 nM), indicating that rapid intracellular entry of ketamine is conserved across species and cellular backgrounds (Fig. 3a–h).

To determine whether the intracellular pharmacokinetic properties observed in immortalized cell lines are preserved in neurons, we expressed PM and cytoplasm-targeted iKetSnFRs in primary cortical neurons and monitored responses during exposure to 500 nM S-ketamine or R-ketamine (Fig. 3i–n). Both enantiomers rapidly entered neurons and generated robust fluorescence responses in both compartments. PM-targeted sensors exhibited rapid fluorescence increases and decay time constants of 542 ± 108 s for S-ketamine and 564 ± 109 s for R-ketamine following washout. Cytoplasmic sensors displayed comparable peak response amplitudes but substantially slower clearance kinetics. Cytoplasmic S-ketamine signals decayed with a time constant of 665 ± 147 s, whereas cytoplasmic R-ketamine signals exhibited markedly prolonged clearance (1130 ± 203 s), approximately twofold slower than observed at the PM despite identical extracellular exposure conditions. These experiments demonstrate that ketamine rapidly permeates neuronal membranes and accumulates within the intracellular space, where clearance is substantially slower than at the PM. Together, these results establish that intracellular ketamine exposure can substantially outlast extracellular drug availability and support the use of iKetSnFRs for resolving compartment-specific ketamine dynamics in living neurons.

### iKetSnFRs reports rapid detection of ketamine in mouse cortical neurons following systemic administration

**Figure 4:**
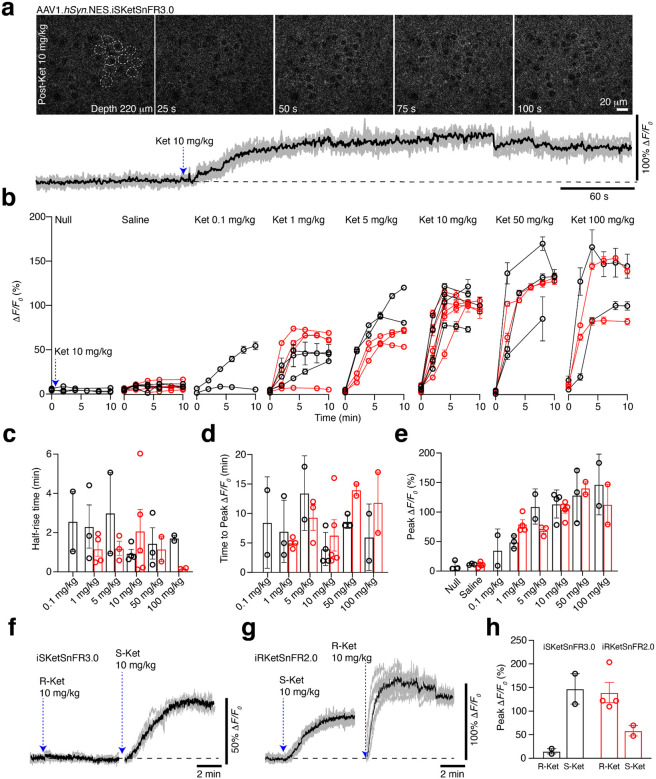
Rapid cortical detection of ketamine in mouse prefrontal cortex following administration. **(a)** Representative two-photon images (top) and fluorescence changes (bottom; individual region-of-interest - ROI - responses in grey, mean response in black) of layer 2/3 neurons expressing cytoplasmic NES-iSKetSnFR3.0 in ACC following i.p. ketamine injection (10 mg/kg; blue dashed arrow). (**b)** Summary of L2/3 neurons expressing Null-iKetSnFR (n = 3), cytoplasmic-iSKetSnFR3.0 (n = 19, black; saline controls n = 3), or cytoplasmic-iRKetSnFR2.0 (n = 16, red; saline controls n = 5) across various ketamine doses (0.1, 1.0, 5, 10, 50, 100 mg/kg i.p.) and saline controls. Each line represents responses from one animal. (**c-e**) Half-rise time, time-to-peak ΔF/F_0_, and peak ΔF/F_0_ across ketamine doses in (b). Null-iKetSnFR + ketamine and cytoplasmic-iSKetSnFR3.0 or iRKetSnFR2.0 + saline showed no response over baseline measurements. (**f)** L2/3 neuronal responses to R-ketamine (R-ket; 10 mg/kg i.p.) followed by S-ketamine (S-Ket; 10 mg/kg i.p.) in an animal expressing cytoplasmic-iSKetSnFR3.0. (**g)** L2/3 responses to S-Ket followed by R-Ket in an animal expressing cytoplasmic iRKetSnFR2.0. (**h)** Summary of average peak ΔF/F_0_ responses to S-Ket and R-Ket in iSKetSnFR3.0 (R-ket: 14.8 ± 5.4, S-ket: 147.1 ± 32.2) and iRKetSnFR2.0 (R-ket: 139.3 ± 21.6, S-ket: 58.5 ± 11.1) animals. Each open circle represents an individual mouse. Bars represent mean ± s.e.m.; all individual animals are shown.

To evaluate iKetSnFR *in vivo* performance in the mammalian brain at cellular resolution, we used adeno-associated virus (AAV) transduction to express cytoplasmically targeted sensor - AAV1.*hSynapsin*.NES.iSKetSnFR3.0 (or iRKetSnFR2.0) - in layer 2/3 neurons of the mouse anterior cingulate cortex (ACC), a prefrontal region. *In vivo* two-photon time-lapse imaging of iKetSnFR signals from neuronal somata revealed rapid, dose-dependent fluorescence increases following intraperitoneal (i.p.) administration of racemic ketamine, with signals emerging within tens of seconds across the tested dose range (Fig. 4a–d). Ketamine dose strongly influenced the magnitude of the fluorescence response but had little effect on rise kinetics (~ 2 min half-rise time) or the timing of peak signal (~ 5–10 min; Fig. 4c–e). The specificity of signals to ketamine presence was confirmed by two controls: a nonbinding control sensor (NES.iNullSnFR; binding site mutated, see Methods) produced no detectable fluorescence changes, and saline injections failed to elicit any response in iKetSnFR-expressing neurons (Fig. 4b, e). When tested with individual enantiomers in the same animals, iSKetSnFR3.0 selectively responded to S-ketamine, whereas iRKetSnFR2.0 detected both R- and S-ketamine, albeit with much lower sensitivity to the latter—demonstrating clear enantiomer specificity of the iKetSnFR sensor family (Fig. 4f–h). Together, these results establish the iKetSnFR sensors as robust and selective tools for quantifying ketamine dynamics *in vivo* across a spectrum of pharmacological regimes, from low (0.1 mg/kg) to antidepressant (10 mg/kg) to dissociative (50 mg/kg) and anesthetic doses (> 100 mg/kg).

### iKetSnFR reveals region-specific cortical ketamine dynamics following intranasal ketamine administration

**Figure 5:**
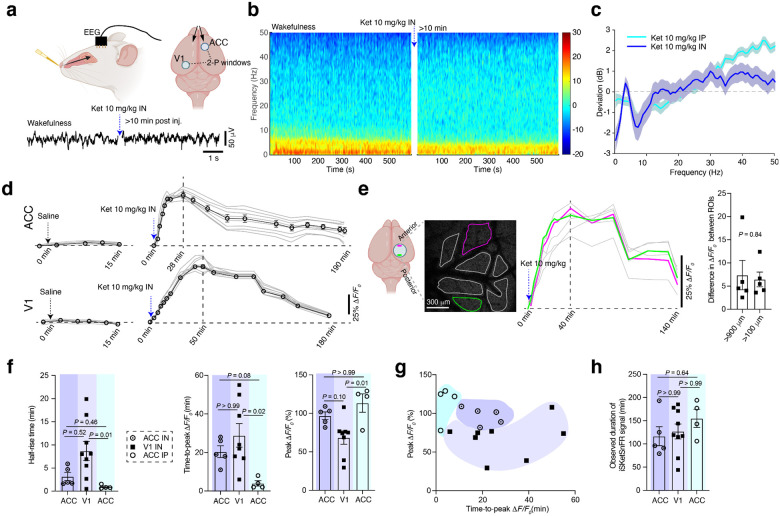
Intranasal ketamine induces rapid cortical desynchronization and sustained, region-specific ketamine dynamics. **(a)** Cartoon of intranasal (i.n.) delivery of ketamine (left) along with EEG and two-photon recording regions (right; ACC - anterior region, V1 - posterior region). **(b)** Time–frequency density spectral array of cortical EEG recorded during wakefulness and following i.n. ketamine (Ket) administration (10 mg/kg). Ketamine induces a brief broadband desynchronization by 10 min. Color scale indicates changes in power (dB). (**c**) Quantification of spectral power deviation across frequencies following ketamine delivered i.n. (blue; n = 4) or i.p. (teal; n = 4). (**d**) Time course of iSKetSnFR3.0 ΔF/F_0_ signals in ACC (top) and V1 (bottom) following i.n. ketamine or saline. Ketamine induces a rapid increase in signal that peaks earlier in ACC than V1 and remains elevated over extended periods. Dashed lines indicate approximate peak times. **(e**) Wide-field iSKetSnFR3.0 imaging through 3-mm cranial windows over V1 (left; peak signal image and representative traces) reveal both rapid and delayed responses. Right, no significant anterior–posterior gradient was observed, as anterior (magenta) and posterior (green) ROIs (spanning >900 μm) exhibited similar fluorescence time courses relative to ROIs < 100 μm. (**f**) Quantification of response kinetics and magnitude across regions following i.n. ketamine (ACC: n = 5; V1: n = 9), compared to intraperitoneal (i.p.) ketamine measured in ACC (n = 4). Left, half-rise time; middle, time-to-peak ΔF/F_0_; right, peak ΔF/F_0_. ACC exhibits faster rise kinetics compared to V1. i.n. ketamine responses in V1 exhibited lower peak amplitudes compared to ACC. Individual animals are shown as points. (**g**) The relationship between time-to-peak and peak ΔF/F_0_ across regions and routes shows increased variability in both temporal dynamics and response magnitude in V1. (**h**) i.n. ketamine produces a duration of detectable signal comparable to that of i.p. ketamine following a single dose. Comparisons were performed using a Kruskal–Wallis test with Dunn’s multiple comparisons *post hoc* test; *P*values are shown. All data are shown as mean ± s.e.m. Each point represents an individual animal.

Intranasal ketamine has been clinically validated as an effective route of administration for treatment-resistant depression^46–49^. Although its therapeutic efficacy and rapid brain entry have been demonstrated—preclinical studies detect cortical ketamine within 2–5 minutes^37,50,51^—the spatiotemporal dynamics of its brain distribution remain unresolved, largely due to the lack of tools capable of capturing real-time PK *in vivo*. To address this gap, we used animals expressing AAV1.*hSynapsin*.NES.iSKetSnFR3.0 to track sensor signals in layer 2/3 neurons (mostly pyramidal cells) following a single intranasal dose of ketamine (10 mg/kg, applied to a single nare) or saline in either the ACC or primary visual cortex (V1) (Fig. 5a). To minimize potential photobleaching and tissue heating during prolonged imaging, data were not acquired continuously over the experimental period. Instead, imaging was concentrated during the rise phase to capture entry kinetics and peak concentrations, followed by intermittent sampling at later time points. Consistent with prior reports^52–54^, electrophysiological (EEG) recordings following intranasal ketamine showed a robust desynchronization of ongoing activity (*e.g*., reduction in delta power and an increase in high-frequency oscillations) within 10 minutes of administration (Fig. 5b). However, these frequency changes were less pronounced than those observed with i.p. ketamine at the same dose (Fig. 5c, **Extended Data** Fig. 4). Across either recording region, two-photon iSKetSnFR3.0 imaging revealed rapid detection of racemic ketamine within tens of seconds, with no signal arising from saline administration (Fig. 5d, **Extended Data** Fig. 5). iRKetSnFR2.0 was also capable of detecting intranasally administered ketamine (**Extended Data** Fig. 5). In the ACC, ketamine elicited a pronounced increase in ΔF/F_0_ that peaked within ~ 20–30 minutes and remained elevated for > 2 hours (Fig. 5d). A similar but temporally delayed and attenuated response was observed in V1, indicating region-specific dynamics in the propagation and persistence of ketamine through the brain. Widefield two-photon imaging through a 3 mm cranial window over V1 (1.2 × 1.2 mm field of view) further confirmed this temporal offset, comparable to that observed in ACC; however, we did not detect significant differences in ketamine signals between anterior and posterior ROIs separated by 900 μm. (Fig. 5e).

Quantification of response kinetics showed that rise time and time-to-peak in ACC following intranasal ketamine were comparable to those observed with i.p. delivery, whereas V1 responses were significantly delayed relative to ACC with i.p. ketamine (Fig. 5f), consistent with preferential early engagement of frontal circuits. Peak ΔF/F_0_ values were similar across regions, indicating that differences in temporal dynamics were not attributable to differences in response magnitude (Fig. 5f–g). Instead, variability in time-to-peak across animals and regions suggests heterogeneous cortical propagation dynamics of ketamine. Notably, iSKetSnFR3.0 signals persisted well beyond the initial rise phase (Fig. 5d, h). Quantification of the duration of detectable signals revealed sustained elevations lasting > 2 hours across regions (Fig. 5h). This prolonged temporal profile was comparable between ACC and V1 despite differences in onset and peak timing, indicating that once engaged, ketamine persists across distributed networks.

### Ketamine persistence in neurons exceeds plasma and EEG-defined kinetics

**Figure 6:**
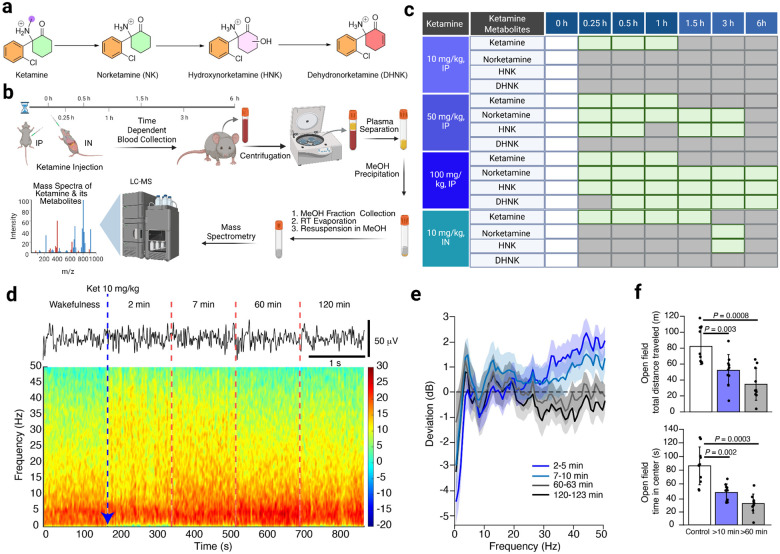
Prolonged brain intracellular retention of ketamine beyond plasma clearance. **(a)** Schematic of ketamine metabolism. Ketamine is metabolized to norketamine (NK), hydroxynorketamine (HNK), and finally to dehydronorketamine (DHNK). **(b)** Experimental workflow for pharmacokinetic measurements. Following ketamine i.p. administration, timed blood collections were performed, plasma was isolated, and ketamine and metabolite concentrations were quantified by LC–MS. **(c)** Heat map summarizing plasma concentrations of ketamine and metabolites following i.p. (10–100 mg/kg) and i.n. (10 mg/kg) dosing. Green shading indicates detectable levels at the indicated time points; gray indicates levels below detection. **(d)** Representative EEG traces (top) and corresponding time–frequency spectrogram (bottom) before and after ketamine IP administration (10 mg/kg). (**e**) Frequency-resolved deviation in EEG power relative to baseline wakefulness at the indicated post-injection epochs, showing normalization in broadband spectral changes at 60 min following ketamine (10 mg/kg IP). (**f**) Open-field behavioral measures following saline (n = 10) or ketamine administration (n = 10 after 10 min, n = 10 after 60 min). Animals exhibit a persistent reduction in total distance traveled (top) and time spent in the center zone (bottom) following ketamine administration. Comparisons were performed using Wilcoxon rank-sum. Bars represent mean ± s.e.m.; individual animals are shown.

We next benchmarked iKetSnFR signals against liquid chromatography-mass spectrometry (LC–MS)-defined ketamine PK following a single ketamine administration to clarify traditional PK versus brain-level signals (Fig. 6a–b). Consistent with prior reports^20,32,33,55,56^, ketamine displayed both rapid uptake (< 15 min) from the i.p. space and metabolism (Fig. 6a, c). After i.p. injection of racemic ketamine (10 mg/kg), LC–MS analysis of plasma collected at multiple time points (t = 0–6 h) revealed that the parent compound (m/z 238.16) was detected only between 15–60 minutes (Fig. 6c). This detection of i.p. ketamine is consistent with the one-compartment extravascular model of ketamine pharmacokinetics (**Extended Data** Fig. 6A). At this dose, the primary phase I metabolites—(R/S)-norketamine (NK; m/z 224.07) and hydroxynorketamine (HNK; m/z 240.13)—from N-demethylation and hydroxylation by cytochrome P450 enzymes were minimal or undetectable. At higher doses (50–100 mg/kg, i.p.), an additional ion at m/z 222.17, corresponding to (R/S)-dehydronorketamine (DHNK), appeared at ~ 30 minutes, indicating further oxidative metabolism of NK, and accumulation of DHNK before elimination. By 60 minutes, HNK and DHNK reached peak abundance as parental ketamine declined sharply, consistent with rapid first-pass metabolism and redistribution^20,56–58^. Ketamine was undetectable after 90 minutes, while NK and DHNK persisted, suggesting longer plasma half-lives and potential accumulation. All metabolites were confirmed by MS/MS fragmentation spectra (**Extended Data** Fig. 6B). Together, these data demonstrate that plasma ketamine is short-lived in rodents, lasting approximately 45–60 minutes after i.p. injection^33^.

We next assessed how ketamine’s EEG signatures and behavioral outputs evolve over the same time window. Acute ketamine (10 mg/kg i.p.) administration produced the expected broadband EEG desynchronization^53,59,60^, but these spectral changes were short-lived, returning to baseline within approximately 60 minutes—mirroring the disappearance of ketamine from plasma (Fig. 6c, d-e). In contrast, behavioral assays revealed a more prolonged functional effect: mice tested in the open field exhibited sustained reductions in locomotor activity and exploratory drive lasting at least 2 hours post-injection (Fig. 6f)^61^. This decrease in exploration was not attributable to dissociation-like behavior or motor impairment, as animals exhibited normal sensory responsiveness on the hot plate test^62^ and maintained normal performance on an accelerating rotarod (**Extended Data** Fig. 7)^63^. Notably, this persistent behavioral state aligned more closely with the prolonged intracellular iKetSnFR signals than with the rapidly declining fast oscillatory EEG activity or plasma ketamine levels.

### iKetSnFRs map subcellular ketamine and reveal persistent retention

**Figure 7:**
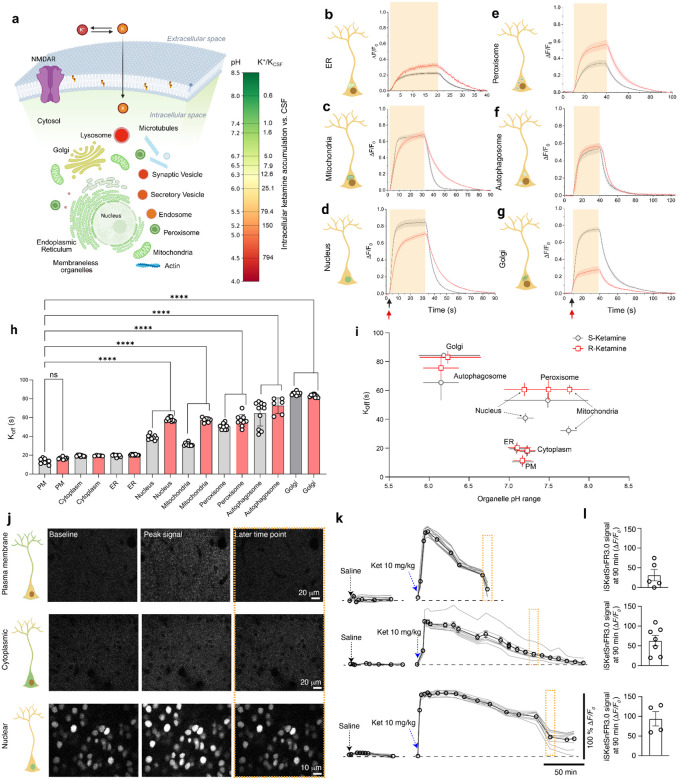
Subcellular compartmentalization and persistence of ketamine accumulation in neurons revealed by organelle-targeted iKetSnFR sensors. **(a)** Schematic model illustrates how ketamine distributes across neuronal subcellular compartments according to organelle pH and membrane permeability. Weakly basic ketamine diffuses across membranes in its neutral form and becomes protonated and trapped within acidic intracellular organelles. The color scale indicates compartmental pH and predicted maximum ketamine accumulation coefficients relative to extracellular cerebrospinal fluid (CSF)^21,24,93^. N-methyl-D-aspartate receptor (NMDAR) shown as purple receptor in plasma membrane. **(b–g)** Representative kinetic traces of organelle-targeted iKetSnFR responses following transient ketamine (200 nM) exposure in Neuro2a cells. Black traces represent iSKetSnFR3.0, and red traces represent iRKetSnFR2.0. Sensors were targeted to the ER (n = 10) **(b)**, mitochondria (n =10) **(c)**, nucleus (n =10) **(d)**, peroxisome (n =10) **(e)**, autophagosome (n =10) **(f)**, and Golgi apparatus (n =10) **(g)**. Note the distinct compartment-specific ketamine uptake and clearance kinetics across organelles, with prolonged retention observed in acidic or trafficking-associated organelles relative to neutral compartments (**Extended Data Fig. 10a-b**). **(h)** Quantification of ketamine washout kinetics [K_off_; time (s)] across subcellular compartments. (**i**) Scatter plot depicting the relationship between organelle pH and ketamine K_off_ measurements [S-ketamine (gray circles) and R-ketamine (red squares)] from compartment-targeted iKetSnFR recordings in Neuro2a cells. Organelles with more acidic luminal pH, including the Golgi and autophagosomes, exhibited the slowest ketamine clearance. **(j)** *In vivo* two-photon images of iSKetSnFR3.0 expression in different neuronal compartments from L2/3 neurons in the prefrontal cortex (top, plasma membrane; AAV1.*hSynapsin*.IgK.iSKetSnFR3.0.PDGFR); middle, cytoplasmic; AAV1.hSynapsin.NES.iSKetSnFR3.0); bottom, nuclear; AAV1.*hSynapsin*.NLS.iSKetSnFR3.0). Baseline, peak ketamine response to 10 mg/kg i.p. injection, time point > 60 min post-injection (orange dashed box). (**k**) Fluorescence changes of L2/3 neuronal responses to saline and ketamine. (**l**) Summary of iSKetSnFR3.0 ΔF/F_0_ responses at 90 min across the different neuronal compartments (cytoplasmic: n = 7, 77.3 ± 11.2; nuclear: n = 4, 106.0 ± 16.0; plasma membrane: n = 6, 37.8 ± 14.2). Each point represents an individual neuron in (h) and animal in (l). All data are shown as mean ± s.e.m. Statistical significance in (h) was assessed by one-way ANOVA with *post hoc* multiple-comparison testing, ****P < 0.0001.

In a final set of experiments, we targeted iKetSnFRs to specific cellular compartments to directly measure compartment-specific accumulation and retention. Above, we demonstrated that both enantiomers produce rapid, concentration-dependent intracellular signals, indicating ketamine has efficient membrane permeation and intracellular access (Fig. 3). Intracellular signals decayed more slowly than those at the PM, demonstrating retention beyond extracellular clearance. Organelle-targeted sensors expressed in cultured Neuro2a cells revealed more pronounced compartment-specific retention with slower rise kinetics than at the PM (Fig. 7a–h). In organelles with neutral pH, iSKetSnFR3.0 ER S-slope was approximately 3-fold lower than that at the PM, suggesting reduced relative accumulation of S-ketamine in the ER compared to the extracellular space (Fig. 7b). In the nucleus, R-ketamine revealed delayed accumulation (20 ± 5 s; P = < 0.001) and prolonged washout (60 ± 6 s; P = < 0.0001), with signals often failing to reach steady state during the 30 s application window (Fig. 7d, h). iKetSnFR sensors targeted to peroxisomes (pH 8.0–8.5) exhibited rise kinetics similar to those observed in the cytoplasm with identical fluorescence responses to both enantiomers (Fig. 7e, h). In organelles with lower pH, such as mitochondria (pH ~ 7.2) and the Golgi (pH 6.0–6.5), targeted sensors exhibited even slower kinetics (Fig. 7g, h). In mitochondria, the fluorescence rise and decay kinetics of R-ketamine (30 ± 5; 60 ± 5 s) were similar to that in the nucleus but substantially slower than that of S-ketamine (10 ± 4; 32 ± 5 s; Fig. 7c, h), indicative of preferential mitochondrial retention. In the Golgi, both enantiomers displayed the slowest off-kinetics (89 ± 6 s; *P* < 0.0001; 64 ± 6 s; *P* < 0.0001), suggesting strong compartmental retention (Fig. 7g–h). Notably, Golgi-localized iRKetSnFR2.0 produced ~ 2-fold lower ΔF_max_/F_0_ values than iSKetSnFR3.0 under matched conditions. Autophagosomes (pH 5.8–6.2)^64^ exhibited a similar rise (~ 20 ± 5 s) and decay (65 ± 5 s, *P* < 0.0001) kinetics (Fig. 7f, h). Therefore, ketamine persists within cells relative to the extracellular space, and subcellular analyses reveal organelle-specific retention suggesting prolonged intracellular exposure [**Table 3; Table 4**].

To determine whether iKetSnFR can resolve ketamine retention at the subcellular level *in vivo*, we monitored fluorescence dynamics across three neuronal compartments using genetically targeted sensors: cytoplasmic (AAV1.*hSynapsin*.NES.iSKetSnFR3.0), nuclear (AAV1.*hSynapsin*.NLS.iSKetSnFR3.0), and plasma membrane–anchored, extracellularly facing (AAV1.*hSynapsin*.IgK.iSKetSnFR3.0.PDGFR) sensors (Fig. 7j). AAV-mediated expression yielded the expected subcellular localization patterns *in vivo* for all three sensors (Fig. 7j), consistent with *in vitro* expression profiles (Fig. 3g, h, **Extended Data Fig. 12**). Two-photon time-lapse imaging of ACC layer 2/3 neurons following racemic ketamine administration (10 mg/kg, i.p.) revealed rapid fluorescence increases across all compartments (Fig. 7j–k). Notably, at 60 minutes post-injection, when ketamine was no longer detectable in plasma measurements, iKetSnFR signals remained persistently elevated in all three locales (Fig. 7j–k). Analyses showed that membrane-localized fluorescence rose and decayed before cytoplasmic and nuclear signals, with nuclear levels rising higher than cytoplasmic and persisting longer. At approximately 90 minutes post-injection, PM-associated signals had declined close to baseline levels (Fig. 7l), while cytoplasmic and nuclear signals persisted > 120 minutes (at 120 min post-inj., cytoplasmic: 21 ± 10.5%; nuclear: 46.5 ± 11.1%). This compartmental hierarchy closely mirrors that observed in primary cortical neurons, where both S-ketamine and R-ketamine exhibited progressively slower clearance from the PM to the cytoplasm and nucleus, with nuclear residence times exceeding membrane-associated clearance by up to four-fold (Fig. 3g, h, **Extended Data Fig. 13**). Taken together, these data suggest that ketamine persists intracellularly *in vivo*, in the cytoplasm and especially the nucleus—much longer than in plasma—which may contribute to its long-lasting antidepressant properties.

## Discussion

This study demonstrates that ketamine enters the brain within 1 minute of administration, rapidly reaches peak levels, and persists for over 2 hours. Notably, this persistence occurs primarily within intracellular compartments, including the cytoplasm and especially the nucleus, rather than in the extracellular space. We developed the genetically encoded fluorescent biosensors iSKetSnFR3.0 and iRKetSnFR2.0 because the molecular mechanisms driving ketamine’s rapid (< 24 h onset) and persistent antidepressant effects remain largely unknown, hindering the rational development of more effective RAADs. Establishing ketamine’s true sites of action first requires a detailed understanding of the biological compartments—brain regions, specific cell types, and individual organelles— that it enters and the time course over which it does so. Yet no existing techniques have been able to measure ketamine’s distribution with adequate spatial and temporal resolution *in vivo*.

To address this challenge, we developed novel genetically encoded fluorescent sensors (iKetSnFRs) that directly and in real-time report ketamine concentrations across subcellular, cellular, and whole-organism scales. iSKetSnFR3.0 (for S-ketamine) and iRKetSnFR2.0 (for R-ketamine) both display robust drug-dependent fluorescence changes. Both sensors exhibit nanomolar-range sensitivity to their cognate ligands. iSKetSnFR3.0 exhibits robust and selective responses to S-ketamine, with a maximal fluorescence change (ΔF_max_/F_0_) of 3.0 and an EC_50_ of 175 nM, whereas responses to the corresponding R-enantiomer and downstream metabolites are markedly attenuated (~ 350-fold specificity for S-ketamine over R-ketamine; Fig. 1d). S-norketamine produces some response, although with substantially reduced potency (EC_50_ = 4.86 μM), indicating that removal of the N-methyl substituent weakens but does not ablate ligand binding. Hydroxynorketamine (HNK) and dehydronorketamine (DHNK) metabolites produce only weak fluorescence responses even at high concentrations, demonstrating that the sensor preferentially recognizes the parent molecular scaffold rather than its major biotransformation products. iRKetSnFR2.0 displays high sensitivity toward R-ketamine, with a ΔF_max_/F_0_ of 3.3 and an EC_50_ of 155 nM (Fig. 1h). Although the sensor retains modest responsiveness to R-norketamine (EC_50_ = 1.08 μM), sensitivity for S-ketamine (~ 30-fold specificity for R-ketamine over S-ketamine) and other metabolites is substantially weaker. Notably, the diminished responses to HNK and DHNK derivatives indicate that subtle stereochemical and oxidative modifications strongly perturb ligand accommodation within the binding pocket. This high *in vitro* selectivity translates well *in vivo*: iSKetSnFR3.0 is unresponsive to R-ketamine *in vivo*, and iRKetSnFR2.0 shows only limited sensitivity to S-ketamine (~ 30% response at 10 mg/kg) relative to R-ketamine (~ 100% response). These properties indicate that both sensors are well-suited for detecting ketamine within pharmacologically relevant concentration ranges with enantiomeric selectivity. Thus, these sensors are ideally suited for addressing critical questions about ketamine biodistribution and PK and can resolve S-ketamine and R-ketamine — both of which are classified as RAADs — with minimal interference from major ketamine metabolites. To our knowledge, this family represents the first set of genetically encoded sensors capable of selectively reporting on both enantiomers of a drug molecule.

Existing methodologies are unable to resolve the earliest moments of ketamine entry into the brain following systemic administration^65,66^. Microdialysis and LC–MS/MS studies provide only minutes-scale sampling, limiting detection to several minutes after dosing, while positron emission tomography (PET) imaging offers frame rates on the order of tens of seconds to minutes and reflects tracer uptake rather than free drug concentrations^66–68^. Electrophysiological or behavioral readouts reveal rapid circuit-level effects but do not directly report pharmacokinetics^69–72^. As a result, the precise time at which ketamine first penetrates the CNS has remained unquantified. *In vivo* two-photon iKetSnFR imaging revealed rapid, dose-dependent (0.1–100 mg/kg) ketamine entry into the mouse cortex (Fig. 4). Upon systemic administration, cytoplasmic iSKetSnFR3.0 and iRKetSnFR2.0 signals in cortical neuronal cells rose within 30–60 s, demonstrating immediate CNS penetration—even without intravenous delivery—a critical observation not previously quantified *in vivo* (Fig. 4a). At 0.1 mg/kg, a small but detectable fluorescence change (ΔF/F_0_ ~5–10%) was observed, which increased at 1 mg/kg (ΔF/F_0_ ~15–25%). Doses of 5–10 mg/kg elicited robust responses (ΔF/F_0_ ~50–100%), while higher concentrations (50–100 mg/kg) saturated the sensors, reaching ΔF/F_0_ ~120–180% (Fig. 4b), consistent with near-maximal occupancy of the sensors by ligand. Across all doses, fluorescence rose rapidly (half-rise ~ 4 ± 2 min) and peaked by ~ 10 ± 4 min, indicating similar ketamine kinetics over the tested range (Fig. 4). Sequential enantiomer delivery revealed distinct but overall comparable dynamics: iSKetSnFR3.0 responded robustly and persistently to S-ketamine with minimal response to R-ketamine, whereas iRKetSnFR2.0 showed strong, slow-decaying responses to R-ketamine and only produced ~ 30% fluorescence response at 10 mg/kg of S-ketamine (Fig. 4e–g). Both sensors showed similar rise times (~ 2 min) and peak amplitudes (~ 100–120% ΔF/F_0_) following i.p. administration, suggesting comparable bioavailability, trafficking, permeability, and intracellular engagement for each enantiomer.

Traditional pharmacokinetic measurements derived from plasma concentrations provide little insight into the spatiotemporal profile of drug exposure at and inside neurons^73^. Consistent with previous reports^20,56,74^, plasma LC–MS measurements demonstrated that ketamine is rapidly absorbed and metabolized, with parent compound concentrations becoming undetectable within ~ 90 min after systemic administration (Fig. 6a–c). In contrast, iKetSnFR imaging revealed that intracellular ketamine exposure persisted for more than 2 hours *in vivo*, with prolonged cytoplasmic and nuclear retention despite declining PM-associated signals (Fig. 5, 7). Following intranasal administration, ketamine also exhibited rapid cortical entry but showed regionally heterogeneous propagation, with persistent intracellular signals extending well beyond the time course predicted by plasma pharmacokinetics (Fig. 5d, h). Together, these findings reveal a striking kinetic dissociation between plasma drug clearance and neuronal drug exposure, suggesting that conventional PK measurements substantially underestimate the duration of ketamine availability within the brain. More broadly, our results introduce neuronal intracellular PK as an additional determinant of drug action that operates alongside receptor affinity, receptor occupancy, and downstream signaling to shape the temporal evolution of circuit responses.

What mechanisms maintain prolonged intracellular ketamine exposure remains an important question. One possibility is that ketamine, a membrane-permeable weak base, undergoes protonation and sequestration within acidic intracellular compartments. To test this hypothesis, we created and validated a broad toolbox of organelle- and membrane-targeted iKetSnFR constructs and validated their targeting by co-expressing them with well-validated markers (Fig. 7, **Extended Data Fig. 11**). Because GFP-based sensors function poorly at pH < 6.0, the most acidic compartment where we could reliably measure was the Golgi, with a pH of 6.3–6.5^24^, and it displayed the slowest ketamine clearance kinetics relative to other organelles (Fig. 7g–i). In previous experiments, S-methadone, another weak base, accumulates ~ 2-fold in the Golgi with respect to PM (extracellular) concentration^42^, and S-ketamine follows this pattern. The unexpected persistence of R-ketamine within the nucleus suggests additional mechanisms that remain to be identified^75,76^. Alternatively, enzymatic or nonenzymatic covalent modification of intracellular proteins by ketamine or its metabolites could further (potentially indefinitely) prolong intracellular residence, analogous to reactions described for other bioactive amines^77–80^. These cultured-cell experiments support compartment-specific intracellular kinetics and reveal differential accumulation of R- and S-ketamine, but they did not reproduce the prolonged retention observed *in vivo*. This discrepancy likely reflects the inability of simplified culture systems to recapitulate the complex physicochemical environment of intact brain tissue, including glial-supported cholesterol homeostasis^81^, restricted extracellular diffusion, organelle pH gradients, liver metabolism, and tissue-scale barriers that collectively shape drug distribution. Future studies will be required to determine the relative contribution of these mechanisms and establish how prolonged intracellular drug exposure influences ketamine’s antidepressant efficacy, toxicity, and abuse liability.

The reasons for the large disparity in ketamine PK between individuals are not clear but may stem from multiple sources. Firstly, the sensor may be expressed differently between individuals, arising from variability in AAV delivery and promoter activity. The *hSynapsin* promoter has been observed to produce heterogeneous expression across neuronal populations, potentially influencing the response amplitude and kinetics^82,83^. Inter-individual differences may also stem from variability in the uptake, metabolism, and receptor signaling of ketamine. We observed the greatest inter-individual variability following intranasal administration, which is expected given several contributing factors. First, intranasally delivered drugs can access the brain through multiple routes, including direct nose-to-brain transport along olfactory and trigeminal nerve pathways, local mucosal absorption followed by blood–brain barrier (BBB) crossing, systemic circulation after vascular uptake, or inadvertent passage with subsequent gastrointestinal or pulmonary absorption^84^. Second, several of these routes—particularly gastrointestinal absorption and systemic delivery—involve first-pass hepatic metabolism, which can substantially delay and reshape brain exposure kinetics^20,85–87^. Third, naturally occurring differences in nasal mucosal permeability and cerebrospinal fluid transport across individuals, influenced by development as well as experimental handling, further contribute to variability. Finally, inter-individual variability in the expression and activity of metabolic enzymes, including cytochrome P450 subtypes, can substantially shape both the extent and temporal profile of ketamine metabolism. For example, expression of CYP2B6—the primary enzyme mediating ketamine metabolism—varies by more than 50-fold across individuals^88^, with substantial differences in hepatic cytochrome expression observed even among inbred mouse littermates^89^. In this context, variability in CYP2B6 and related metabolic pathways, together with differences in vascularization and tissue-level drug delivery, likely contributes to the heterogeneous ketamine dynamics observed across animals. Future studies employing cell type–specific imaging strategies or intranasal absorption enhancers^90–92^ will help disentangle these sources of variability, enabling a more complete understanding of how ketamine is delivered into the CNS and how it modulates neural activity across distinct cell populations *in vivo*.

## Methods

### Iterative site-saturated mutagenesis to produce iKetSnFR

The original protein from which the iKetSnFRs are derived was a biosensor named AK1, previously described^40^. AK1 itself was the result of a Y357G mutation to cc90, an acetylcholine biosensor^40,94^. Y357 was specifically chosen for mutagenesis, as it is in close proximity to the secondary amine group of S-ketamine when it is computationally docked within the binding pocket of the periplasmic binding protein (PBP). From AK1, we chose to mutate F457 due to its location within the PBP hinge, which undergoes a large ligand binding-dependent Venus flytrap conformational change. Further, Phe is hydrophobic and aromatic, which we postulated could inhibit ligand-binding-dependent movement of the hinge. This resulted in iSKetSnFR1.0. Next, we aimed to mutate G395, also in the PBP hinge. Gly is neutral, and we had thought that a charged amino acid could result in increased ligand binding. In addition, Gly is flexible, and replacing it may stabilize the ligand-bound conformation. The next site of interest was I10, which is near the N-terminal hinge and lies within the binding pocket. Following the same reasoning, we assumed this mutation would modulate hinge dynamics and increase transmission of ligand binding-driven changes in fluorescent signal. These mutations resulted in iSKetSnFR2.0. As we were developing the S-ketamine and R-ketamine sensors in parallel, we noticed that mutating T360 improved the sensitivity of R-ketamine for iRKetSnFRs. Thus, we wondered if this mutation may also be beneficial for our iSKetSnFR sensors, especially as it sits just above the superior aspect of the binding pocket. L359 is similarly in an adjacent position; mutation of this residue led to iSKetSnFR3.0.

For iRKetSnFRs, we similarly began with AK1. We first mutated H68, which was predicted from computational docking to make a cation-pi interaction when the histidine is protonated. This mutation likely has a role in stabilizing charges on ketamine’s cyclohexanone ring. Thus, as R-ketamine is an enantiomer of S-ketamine, we wondered if weakening this cation-pi interaction would result in increased response to R-ketamine specifically. Next, we targeted E43, in both the binding pocket and the hinge. As mentioned above, we were at this point performing site-saturated mutagenesis in parallel for both iSKetSnFRs and iRKetSnFRs. Thus, we also mutated I10, which resulted in improvement. After determining the crystal structure of iSKetSnFR2.0, we felt A435 was an important site, as it is adjacent to W436, one of the three aromatic residues that are known to interact strongly with S-ketamine from our prior docking and mutagenesis experiments^40^. Next, F12 was targeted as it is firmly within the binding site, and we felt it would improve EC50 if we were able to replace Phe with a more polarizable pi system and, therefore, make stronger cation-pi interactions with ketamine’s tertiary ammonium. This work resulted in iRKetSnFR1.0. From here we addressed A360, which is very close to G357, again a residue that likely makes strong cation-pi interactions. We were also interested in increasing ΔF_max_/F_0_. Thus, we mutated P324, which is in Linker2 (the linker from cpGFP to PBP); we postulated this may influence ligand binding-driven conformational changes. Lastly, we targeted L359 following the same logic as for iSKetSnFRs. This resulted in iRKetSnFR2.0.

We designed three sets of forward primers and one reverse primer per targeted SSM location, which were synthesized (Integrated DNA Technologies) and then concentrated via butanol precipitation (to filter out shorter truncated sequences) as pellets before being resuspended in nuclease-free water. We then used a spectrophotometer (NanoDrop ND-1000 UV-Vis, Thermo Fisher Scientific) to measure the concentration of each primer solution, after which the three forward primers for each target site were diluted to a concentration of 100 μM. These were then mixed in the aforementioned 12:9:1 ratio, after which this mixture was diluted to a final concentration of 5 μM, the same as the reverse primer. To perform PCR amplification, we first made a master mix that contained 1 μL of parent iKetSnFR plasmid at 60 ng/μL, 10 μL of buffer (Phusion 5X HF Buffer, New England Biolabs), 1 μL of free DNA bases (dNTPs; dNTP Solution Mix, NEB), and 33.5 μL of nuclease-free water. Next, 46 μL of the master mix was split into 1.5 mL microcentrifuge tubes (Eppendorf), to which was added 2 μL of the forward primer mix. Each 48 μL mix was split into PCR tubes (USA Scientific) with two tubes (24 μL) for every SSM site. For this first PCR, the denaturation temperature was 98°C (50 s), the annealing temperature was 57°C (30 s), and the extension temperature was 72°C (5 min) over four total cycles. After the PCR plate warmed to its denaturation temperature, the program was paused to add polymerase (0.25 μL per tube; Phusion HF DNA Polymerase, NEB) as the plate temperature exceeded the specific primer annealing temperature (usually 55–60°C); this ‘hot start’ method helps prevent nonspecific primer extension at lower temperatures^95^. After polymerase addition, four cycles with just the forward primer mix were run to bias the final PCR product toward the 67 possible mutant sequences rather than the parental sequence.

Next, the two tubes for each SSM were combined, and 2 μL of reverse primer was added. PCR was then performed with the same conditions as above, except that the denaturation time was 40 s for 16 total cycles. After the second PCR, the two tubes for each SSM were combined and run on a 0.8% agarose gel with ethidium bromide (EtBr) dye (to visualize DNA) in Tris-acetic acid-EDTA (TAE) buffer against a 1 kilobase pair (kbp) ladder. The ~4.4 kbp fragments were excised, and the DNA was purified following a gel extraction protocol (QIAquick Gel Extraction Kit, Qiagen). After measuring the concentration of the resuspended plasmid DNA, we performed an overnight digest in a 37°C incubator using *Dpn*I (NEB), which digests methylated DNA (thereby selectively digesting parental sequences, as newly synthesized DNA is not methylated^43,95^.)

The next day, the *Dpn*I-digested SSM product was eluted via PCR clean-up (QIAquick PCR Purification Kit, Qiagen) to remove the enzyme and other digestion byproducts. The SSM mix was then electroporated into *E. coli* TOP10 cells as previously described and plated for overnight growth. The next day, five colonies per SSM plate were picked and each grown in 5 mL of LB with 5 μL of ampicillin at 37°C overnight for ~18 h, after which the cultures were spun down, the supernatant pulled off, and the pellets miniprepped (QIAprep Spin Miniprep Kit, Qiagen) before being sent for sequencing (Laragen) to confirm that there was a high proportion (4 or 5 out of 5) of mutated sequences at the SSM site among the picked colonies. Once confirmed, the plate was washed with 5–10 mL of LB (combining all of the individual colonies into one suspension), and that solution was miniprepped as described previously; after determining the concentration via spectrophotometry, 300 ng of the plasmid miniprep mix was transformed into chemically competent *E. coli* BL21(DE3) cells via a 30 s heat shock at 42°C, incubated in a 37°C water bath for 1 h, and plated as previously described for overnight growth in a 37°C incubator.

The next day, colonies were picked from the plate, with one seeded into each well of a 96 well deep-well plate with 800 μL of ZYP 5052 media (to improve yield via unblocking *lacZ*^96^; the plate was then covered in cheesecloth and placed in an incubator shaker set to 30°C and 250 rpm for 24 h.

The next day, a replica plate was made by pipetting 50–80 μL of each culture into the corresponding well of a 96-well assay plate, which was covered and placed in a −80°C freezer to be thawed when a promising candidate mutation had been identified; the original SSM plate was spun down, the supernatant pulled off, and the pellets stored in a −80°C freezer. When ready, the original SSM plate was thawed at RT and the pellets were resuspended in 800–1000 μL of 1x PBS, pH 7.0. The plate was then subjected to one round of liquid nitrogen freeze-thaw, after which it was spun down as described above.

Next, 200 μL of supernatant was withdrawn from each well. 100 μL was dispensed to test ketamine-induced fluorescence changes in the corresponding well of one 96-well assay plate, and 100 μL was dispensed to test choline-induced fluorescence (to measure background responsivity to choline) in the corresponding well of another 96-well assay plate. Two stock solutions of R-ketamine and S-ketamine were made in 3x PBS, pH 7.0 with appropriate pH adjustments. Stock solutions were made such that the addition of 10 μL of R-ket or S-ket to each well containing 100 μL of lysate resulted in a final concentration of the particular enantiomer equal to that which produced ΔF/F_0_ = 1 relative to the parent plasmid. The ketamine solution was loaded into the injector of an automated 96-well plate reader; 10 μL of ketamine was then added automatically to each well, the plate was shaken, and fluorescence measurements were taken as described previously. The injector was then rinsed, and the process was repeated for the choline solution added to a second lysate plate. After the data was exported, the ratio of the ketamine-induced fluorescence to the choline-induced fluorescence of each corresponding well was calculated.

After assessing the ratios, the best mutants (ΔF/F_0_ > 1.3, or a 30% increase over choline^43^) were then selected for further analysis, the first step of which was sequencing to confirm that the candidates were not identical to the parent plasmid. Each nonparent mutant was then inoculated from the corresponding replica plate well, with 1.5 μL pipetted into 10 mL LB with 10 μL ampicillin and grown in a 37°C incubator shaker for ~18 hours. The next day, the cultures were miniprepped, and the resuspended DNA was quantified via spectrophotometry to determine if the concentrations were high enough (~>15 ng/μL) before being sent out for sequencing. Once the absence of the parent sequence was again confirmed, 100 ng of mutant DNA was transformed into *E. coli* BL21(DE3) cells and plated for overnight growth in a 37°C incubator as before. The next day, mutants were pelleted and lysed as described above, then assessed with full concentration-response curves against R-Ket and S-Ket plates as previously described. After the best-performing mutant (highest S-slope) was identified, this mutant was carried forward into the next round of SSM; additional rounds following these protocols were performed until the EC_50_ in lysate was within the pharmacologically relevant concentration range; the ΔF_max_/F_0_ value was typically 2–8, with higher values preferred due to the decrease in maximal fluorescence when the sensor was expressed in cells/neuronal cultures and *in vivo*.

### Purifying iKetSnFR protein

After settling on new iKetSnFR sequences, we next generated purified protein for use in further characterization^40^. Compared to lysate, purified protein contains fewer contaminants and more faithfully reports the performance of the sensor in response to the ketamine enantiomers. First, the new iKetSnFR plasmid was transformed into BL21(DE3) cells, incubated, and plated overnight as previously described. The next day, one colony was picked for growth in 100 mL of LB along with ampicillin, MgSO_4_ (for stabilization of DNA), ZYP-5052 media, and extra salts; the flask was loosely covered to allow for aeration and placed in a 30°C incubator shaker (250 rpm) for 24–30 h. Once ready, the contents appeared greenish-yellow; the mixture was then transferred to two 50 mL tubes to be spun down in a centrifuge as previously described. The supernatant was pulled off, and the pellets were gently rinsed several times using 1x PBS, pH 7.0 before being placed in a −80°C freezer overnight (or for long-term storage). When beginning the purification process, the frozen pellets were thawed on ice and resuspended in 5 mL of 1x PBS, pH 7.4. The cells were then sonicated (13% amplitude, 0.7 s time on, 0.2 s time off, 30 s total) 3–6 times with 3 min in between on ice to increase lysis efficiency. After another round of centrifugation, the supernatant was pulled off and passed through a 0.2 μm filter (Syringe Filter, VWR) to remove additional debris and unlysed bacteria before being injected onto a pre-washed Ni-NTA column in a fast protein liquid chromatography (FPLC) system (ÄKTA Start, Cytiva). The SnFR protein was eluted into glass vials using an imidazole gradient (20–200 mM in 1x PBS, pH 7.4 solution); the bacterial expression vector contains a His_6_ tag, which interacts with the Ni^2+^ ions on the column at lower concentrations of imidazole and was washed off at higher concentrations. To make sure the purity of the protein fractions was high (> 95%) and that the molecular weight of the protein eluted was correct, we diluted 7.5 μL samples of representative fractions from across the ~25 generated (usually 11 total) in 7.5 μL of glycerol-based buffer (4x Laemmli Sample Buffer, Bio-Rad) mixed with β-mercaptoethanol (BME). After denaturing the proteins at 95°C for 5 min, we injected each into separate lanes of a gel in Tris-glycine buffer alongside a ladder (10–250 kDa, 70 Precision Plus Protein Dual Color Standards, Bio-Rad). After ~30 min at 200 V, we removed the gel and placed it inside of a chamber filled with dye (Coomassie brilliant blue, Bio-Rad) on a shaker table overnight for staining. After rinsing the gel in deionized water several times the next morning, we examined the purity of the fractions. Once purity was confirmed, all fractions (excluding the first fraction) with significant protein content were combined and concentrated via centrifugation (8 minutes at 2000 rpm and 4°C) in a filter unit (Amicon Ultra-15, 30 kD cutoff, Millipore). Once concentrated to approximately 500 μL, the protein was buffer exchanged into 3x PBS, pH 7.0 before being read on a spectrophotometer to determine its A280. The final concentration (C, in M) was determined via Beer’s Law, A = *CεI*, where A is the absorbance at A280, ε is the molar attenuation coefficient (specific to each protein, in units of M^−1^cm^−1^), and l is the path length (equal to 1 cm). The final step involves testing the purified protein. The ladder (left) was used to measure the size of the proteins (in this case, approximately 62 kDa). The presence of extra bands in a fraction or less-resolved bands disqualifies it from inclusion in the pool of protein later used for *in vitro* concentration-response experiments against the aforementioned ketamine plate. The first three rows of a 96 well assay plate were filled with 50 μL of 200 nM purified protein using the automatic pipette system, which was then used to create a mixture between sensor and ligand from the ketamine plate as previously described. The plate was then placed in the automatic plate reader and the Ket-induced fluorescence response measured as previously described. Concentration-response curves were generated as before. All concentration-response experiments using purified iKetSnFR protein were performed in triplicate, and the standard deviation was calculated for each concentration.

### Characterizing purified iRKetSnFR and iSKetSnFR proteins

Isothermal titration calorimetry (ITC) data were obtained by preparing two solutions, both in 3x PBS, pH 7.0: purified iRKetSnFR or iSKetSnFR at a concentration at least 10 times the purified protein EC_50_; and ligand (R- or S-ketamine) at a concentration 10 times above the SnFR concentration. These solutions were then loaded into the injection syringe and sample cell, respectively, of the ITC system (Affinity ITC, TA). The volume of each injection was held constant and was chosen based on estimation protocols within the proprietary software (NanoAnalyze, TA). The experiment was run at room temperature (RT), and both the syringe and the cell were rinsed with 3x PBS, pH 7.0 before and after the experiment. All solutions were degassed and inspected to prevent bubbles from forming in the cell. Experiments were performed in triplicate, and the mean and standard error of the mean (SEM) were calculated for each thermodynamic parameter (Fig. 2e–g).

### Targeting iKetSnFRs to Mammalian Cells and Subcellular Compartments

For expression in mammalian cells, the iKetSnFR recombinant plasmids were cloned into pCMV(MinDis), a variant of pDisplay (Thermo Fisher Scientific). This plasmid is well-suited for expression on the plasma membrane (PM), as it contains a C-terminal Myc tag (to help detection of recombinant proteins), an N-terminal immunoglobulin kappa light chain (IgK) secretion leader sequence (to target the SnFR to the secretory pathway), and a C-terminal platelet-derived growth factor receptor (PDGFR) transmembrane helix (to help anchor the SnFR to the PM facing outward); a hemagglutinin (HA) tag was removed, as it interfered with the overall fluorescence response. To target the ER, the Myc tag was removed, and the 14 C-terminal AAs were replaced with an ER retention motif (QTAEKDEL) [**Table1; Table2**].

To synthesize both the PM- and ER-targeted versions, we employed circular polymerase extension cloning, which relies on engineering overlapping regions at the termini of both an insert sequence and a vector sequence; each uses the other as a template for extension during PCR, with leftover nicks repaired during bacterial transformation. First, we used PCR to extend the new recombinant plasmid sensor (the insert) to include overlapping regions with a vector sequence taken from the backbone of an already functional mammalian plasmid targeted to either the ER or the PM. This step required a master mix for four total PCRs (insert for ER, insert for PM, vector for ER, vector for PM) composed of 35.5 μL nuclease-free water, 10 μL buffer, and 1 μL dNTPs, which was then split into 46.5 μL aliquots.

Next, specific primers were added (1 μL each of forward and reverse) to every reaction to either engineer overlapping regions (the two inserts had separate forward primers and the same reverse primer) or to remove an old iSKetSnFR sequence to amplify the mammalian vector (the two backbones had the same forward primer and separate reverse primers) with identical overlapping regions. The insert plasmid (1 μL) was then added to the two aliquots with primers for extending the insert; the ER vector plasmid (1 μL) was added to the aliquot with primers to harvest the ER backbone, and the PM vector plasmid (1 μL) was added to the aliquot with primers to harvest the PM backbone. The total volume in each aliquot was then split between two PCR tubes for each of the four reactions before polymerase was added during a “hot start” as described previously and run for 20 cycles using PCR conditions described earlier.

Once the run was completed, the four PCR products were added to the wells of an agarose gel as described previously, after which the proper bands (inserts: ~1.8 kbp; backbones: ~3.5 kbp) were cut out, the DNA was eluted via gel extraction, and the four PCRs were digested overnight with *Dpn*I as described before. The next day, we performed a PCR clean-up as described previously and quantified the concentrations using a spectrophotometer as described earlier before preparing for the CPEC reaction (we expected 20–50 ng/μL)^41^.

We therefore prepared two PCR mixtures: 10 μL insert (either ER or PM), 10 μL backbone (either ER or PM, matched to the insert), 1 μL dNTPs, 10 μL buffer, and 18.5 μL nuclease-free water. The mixtures were split into PCR tubes, and polymerase was added via another “hot start”; the PCR was run using settings described previously. Once the PCR was completed, we performed another PCR cleanup to elute each of the two completed plasmids (ER.iKetSnFR and PM.iKetSnFR), which were then transformed into TOP10 via electroporation as described previously and added to ampicillin plates incubated overnight at 37°C for 18 h.

The next day, we picked five colonies per plate to grow in 5 mL LB and 5 μL ampicillin overnight in an incubator shaker at 37°C and 250 rpm for 24 h. The following day, we centrifuged the 10 total cultures before miniprepping each of them for sequence analysis. Once the sequences had been confirmed, the plasmids were ready for use in mammalian cells^41^.

We generated a series of additional mammalian vector plasmids targeted to various subcellular compartments by using specific primers to add and delete the sequences listed below; all PCR conditions were as described previously. Except for the cytosol-targeted constructs, only the iKetSnFRs were modified to target these other subcellular compartments. To generate iKetSnFRs targeting the cytosol and nucleus, we removed the ER retention motif from the ER-targeted plasmid as well as the N- and C-terminal tags via PCR. For cytosolic expression, we used PCR to add a nuclear exclusion signal (NES; DIDELALKFAGLDL) at the N-terminus after the start codon. To localize the iKetSnFR to the nucleus, we used PCR to add a nuclear localization signal (NLS; PKKKRKV) to the C-terminus [**Table1; Table2**].

Next, we made additional changes to localize iKetSnFRs to the peroxisome and mitochondria. For targeting the peroxisome, we inserted a peroxisome localization sequence (SKL) at the C-terminus of the cytosol-targeted construct. To target mitochondria, we added the COX-VIII tag to the cytosol-targeted plasmid just downstream of the NES; COX-VIII is a duplicated mitochondrial localization sequence derived from the VIII subunit precursor of human cytochrome c oxidase, which we ordered as a synthetic gene from IDT [**Table1; Table2**].

Finally, to target the remaining compartments of interest, we used Gibson assembly, an approach that leveraged the presence of overlapping ends shared by at least two (but occasionally several) DNA fragments to perform an exonuclease-mediated single-step ligation. We added an exonuclease, a polymerase, and a ligase to a reaction mixture containing 0.02–0.5 pmol total fragments and 10 μL master mix (NEB); in a single 30-min step at 50°C, the exonuclease removed bases from the 5’ end, the fragments annealed, the polymerase filled in remaining gaps, and the ligase joined the segments together.

To make the common starting sequence for the remaining plasmids, we first digested a cytosol-targeted iKetSnFR construct using the restriction enzymes *EcoR*I and *Bgl*II overnight in a 37°C incubator. We then ran the double-digest product on a gel and purified it using gel extraction as described previously.

For targeting the Golgi apparatus, we purchased the synthetic fragment [IgK]-[B4GALT1]-[KDPPVAT] (B4GALT1: β−1,4-galactosyltransferase 1) with overhanging ends (Twist Bioscience); the IgK leader sequence targeted the secretory pathway as mentioned previously, and B4GALT1 had an N-terminal sequence that localized the protein to the Golgi^97^; the last sequence was a linker. After PCR amplification of the synthetic fragment, we used Gibson assembly to ligate the synthetic gene to the double-digested backbone, as described previously, to create the final Golgi-targeted construct^42^ [**Table1; Table2**].

The same strategy was employed to assemble the constructs targeted to the autophagosome, with the only difference being the middle targeting sequence (microtubule-associated protein light chain 3; LC3). For F-actin localization, we likewise purchased a synthetic fragment containing [NES]-[Lifeact], the second sequence being a small peptide tag for actin; this was PCR amplified for Gibson assembly with the same backbone as described above [**Table1; Table2**].

We used two different mammalian cell lines for our *in vitro* experiments: Neuro2a cells and SH-SY5Y cells. Neuro2a is an immortalized cell line derived from a mouse neuroblastoma. They have a doubling time of ~10 h and grow as a monolayer of round, loosely attached cells (average diameter 16 μm). SH-SY5Y is an immortalized cell line derived from human neuroblastoma cells that has the advantage of being both human-derived and brain-derived. These cells have a doubling time of 27–67 h and grow smaller as confluence increases (from 43 μm to 17 μm diameter).

### Plasmids developed for cellular imaging

**Table:** 

Constructs	Source or reference	Identifier	Additional Information
**Biosensor plasmids for S-Ketamine**			
iSKetSnFR3.0	This paper	Addgene: XXX	For bacterial protein expression
pCMV(MinDis)-PM.iSKetSnFR3.0	This paper	Addgene: XXX	Plasma membrane
pCMV(MinDis)-ER.iSKetSnFR3.0	This paper	Addgene: XXX	Endoplasmic reticulum
pCMV(MinDis)-Cyto.iSKetSnFR3.0	This paper	Addgene: XXX	Cytoplasm
pCMV(MinDis)-Peroxisome.iKetSnFR3.0	This paper	Addgene: XXX	Peroxisome
pCMV(MinDis)-Mito.iSKetSnFR3.0	This paper	Addgene: XXX	Mitochondria
pCMV(MinDis)-Nucleus.iSKetSnFR3.0	This paper	Addgene: XXX	Nucleus
pCMV(MinDis)-Golgi.iSKetSnFR3.0	This paper	Addgene: XXX	Golgi
pCMV(MinDis)-LC3.iSKetSnFR3.0	This paper	Addgene: XXX	Autophagosome
pCMV(MinDis)-F-Actin.iSKetSnFR3.0	This paper	Addgene: XXX	F-Actin
AAV1-*hSyn*-Cyto.iSKetSnFR3.0	This paper	Addgene: XXX	Cytoplasmic version for neuronal expression
AAV1-*hSyn*-PM.iSKetSnFR3.0	This paper	Addgene: XXX	PM version for neuronal expression
AAV1-*hSyn*-Nucleus.iSKetSnFR3.0	This paper	Addgene: XXX	Nuclear version for neuronal expression
**Biosensor plasmids for R-Ketamine**			
iRKetSnFR3.0	This paper	Addgene: XXX	For bacterial protein expression
pCMV(MinDis)-PM.iRKetSnFR3.0	This paper	Addgene: XXX	Plasma membrane
pCMV(MinDis)-ER.iRKetSnFR3.0	This paper	Addgene: XXX	Endoplasmic reticulum
pCMV(MinDis)-Cyto.iRKetSnFR3.0	This paper	Addgene: XXX	Cytoplasm
pCMV(MinDis)-Peroxisome.iRKetSnFR3.0	This paper	Addgene: XXX	Peroxisome
pCMV(MinDis)-Mito.iRKetSnFR3.0	This paper	Addgene: XXX	Mitochondria
pCMV(MinDis)-Nucleus.iRKetSnFR3.0	This paper	Addgene: XXX	Nucleus
pCMV(MinDis)-Golgi.iRKetSnFR3.0	This paper	Addgene: XXX	Golgi
pCMV(MinDis)-LC3.iRKetSnFR3.0	This paper	Addgene: XXX	Autophagosome
pCMV(MinDis)-F-Actin.iRKetSnFR3.0	This paper	Addgene: XXX	F-Actin
AAV1-*hSyn*-Cyto.iRKetSnFR2.0	This paper	Addgene: XXX	Cytoplasmic version for neuronal expression
AAV1-*hSyn*-PM.iRKetSnFR2.0	This paper	Addgene: XXX	PM version for neuronal expression
AAV1-*hSyn*-Nucleus.iRKetSnFR2.0	This paper	Addgene: XXX	Nuclear version for neuronal expression

### Cell culture

To begin a cell culture, a vial of cells (ATCC) was thawed and passaged three times using recommended practices (ATCC). We used Eagle’s minimal essential medium (EMEM) (Thermo Fisher Scientific) for SH-SY5Y cells or Dulbecco’s modified Eagle’s medium (DMEM) (Neuro2a) supplemented with 10% fetal bovine serum (FBS) (Thermo Fisher Scientific) and 1% penicillin-streptomycin (Thermo Fisher Scientific) as the growth media. Trypsin (ATCC) was used to lift the cells during passaging, which was carried out every 3–5 days; after ≤ 20 passages, the cells were bleached, and a new line was initiated. Cells were stored in an incubator set at 37°C with 5% CO_2_ in 35-mm dishes containing a 14-mm coverslip (MatTek).

For transfection of mammalian cells, we used Lipofectamine 3000 (Invitrogen). To prepare for imaging experiments, approximately 100,000 HeLa cells or ~50,000 Neuro2a and SH-SY5Y cells were plated in 35-mm dishes and incubated for 24 h under the conditions described above. The cells were then transfected with a mixture containing 0.25–1 μL of plasmid at 500 ng/μL, twice that volume of P3000 reagent (Invitrogen), and three times that volume of Lipofectamine 3000; Opti-MEM (Thermo Fisher Scientific) was added to bring the total mixture volume to ~0.5 mL. This mixture was added to the dishes along with 1.5 mL of Opti-MEM. Transfection occurred within 24 h, after which the dishes were either used for imaging or exchanged into EMEM for an additional 24 h of growth.

### Primary mouse cortical co-culture

#### Solutions and Preparation:

Dissection Media (DsM) was prepared by dissolving 5 mL Pen/Strep (#P4333, Millipore Sigma), 5 mL of 100 mM Sodium Pyruvate (11360070, Gibco), 10 mL HEPES, and 5 mL 45% Glucose (in ddH_2_O) in 500 mL 1X HBSS [[-]Ca, [-]Mg] (55037C, Millipore Sigma) and filtering through a 0.22 μm filter. Papain solution was prepared by dissolving PDS Kit (LK003176, Worthington Biochem) in 5 mL of 1X HBSS [-Ca, -Mg] to yield a solution containing 20 units of papain/mL, 1 mM L-cysteine, and 0.5 mM EDTA. Neuronal Media (NM-0) was prepared by dissolving 5 mL Pen/Strep, 5 mL 100X Glutamax (35050061, Thermo Scientific), and 5 mL of 50X 2% heat-activated B-27 Supplement (17504044, Thermo Scientific) in 500 mL of Neurobasal Medium [[-]-L-Glutamine] (21103049, Gibco).

#### Dissection and Dissociation:

Primary cortical co-cultures were prepared from the cortices of a single litter of up to six C57BL/6J pups (P0-P1) to yield neuron-glia co-cultures (~85% neurons, ~10% astrocytes, ~3% microglia). Pups were anesthetized on ice, then sequentially decapitated and dissected to harvest the cortices in DsM. Cortical tissue was then transferred to a 50 mL conical tube of pre-warmed papain solution in addition to 25 μL of RNase-free DNase (89836, Thermo Scientific) and placed in a 37°C bead bath for 15 minutes (gently agitated every 2–3 minutes). After 15 minutes, 10 mL of pre-warmed DsM was then added to the papain solution and incubated at 37 °C for an additional 5 minutes. The papain solution was then gently decanted such that ~5 mL of liquid remained to which 15 mL of pre-warmed NM-5 was added. Tissue was then triturated with pipettes of decreasing diameter (3–4 each), first starting with a 5 mL serological pipette and ending with the second Pasteur pipette (medium diameter). The mixture was then carefully poured through a 70 μm cell strainer (352350, Falcon) into a 50 mL tube. After gently swirling to disperse cells, cell concentration was calculated using the Cellometer Auto 2000 (26407, Nexcelom Bioscience). The solution was then triturated with the smallestdiameter pipette and diluted with pre-warmed NM-5 to the desired plating density. Media was changed ~24 hours later with maintenance media (NM-0).

#### Viral Transduction:

Cultures were transduced with AAV vectors at DIV 6–7 at a target Multiplicity of Infection (MOI) of 10,000. AAV vectors were diluted in NM-0 prior to media change and allowed to incubate for 48 hours before an additional media change.

### Cellular iKetSnFR imaging

To image transfected mammalian cells during concentration–response experiments, we used the same apparatus and conditions described for our experiments with iNicSnFR and iSKetSnFR2.0^39,40^. Dishes were placed in a perfusion ring (DH 40i, Harvard Apparatus) supported by a stage adapter (SA-TS100, Warner Instruments) under an inverted widefield epifluorescence microscope (IX-81, Olympus) equipped with a 40× oil-immersion objective (NA = 1.0). Images were captured with a back-illuminated EMCCD camera (iXon DU-897, Andor) using vendor software (Andor IQ2, Andor) at 3–4 frames per second. We installed two light-emitting diodes (LEDs), 470 nm and 400 nm (LZ1–10DB00, LED Engin), operated with currents between 40–800 mA and paired with a 40-nm bandpass filter centered at 480 nm (ET 470/40X, Chroma Technology). Solutions flowed into the dish via gravity from elevated reservoirs controlled by solenoid valves (Automate Scientific) that converged into a common manifold, delivering flow rates of 5–6 mL/min. All drug solutions were prepared in HBSS to mitigate pH changes that would otherwise elevate the F_0_ of iKetSnFRs and artificially restrict ketamine-evoked fluorescence changes; we used Teflon tubing (Versilon, McMaster-Carr) with a fluorinated ethylene propylene lining for the same purpose. Solution inflow and outflow occurred through stainless-steel tubes positioned 4 mm apart, with a vacuum aspirator connected to a trap to pull solution across the monolayer and establish laminar flow. We still observed small fluorescence artifacts from buffer changes, probably due to small (<0.1) changes in pH affecting sensor fluorescence.

Imaging data were analyzed offline using the ImageJ *Time Series Analyzer* plug-in. The brightest cells—those that often saturated pixels even at baseline—were excluded because their fluorescence responses exceeded the detector’s dynamic range. Regions of interest (ROIs) were selected based on the construct being imaged. For iKetSnFRs targeted to intracellular compartments (e.g., ER, peroxisome), ROIs were drawn around the entire fluorescent region, while for iKetSnFRs targeted to membranes (e.g., PM, F-actin), ROIs were drawn only around the cell perimeter. ΔF/F_0_ time-series traces were produced by first subtracting the background (extracellular) signal and then calculating the difference between the first 3 s of the time series and all subsequent frames. We then used analysis software (Origin 2018) to plot the traces, correcting for baseline drift using a spline toolbox. Traces from a minimum of five cells (across at least two fields of view, FOVs) were averaged and displayed with the standard error of the mean (SEM) shown as semi-transparent shading.

Visualizing iKetSnFR expression in subcellular compartments or at the plasma membrane required higher resolution than was achievable with the 40× objective described above. Thus, we also imaged compartment-targeted sensors using a motorized spinning-disk laser-scanning confocal inverted microscope (Eclipse Ti-E, Nikon) equipped with a 100× oil-immersion objective (NA = 1.49; working distance = 120 μm) and a 488-nm laser at 15% power. The microlensing of the spinning disk and the IMCCD camera allowed low-light imaging, reducing photobleaching and extending viable imaging duration. Imaging took place in a custom incubator (Okolab) maintained at 37°C and 5% CO_2_. Initial images were acquired with cells in HBSS alone; to induce fluorescence, we doubled the bath volume by adding ketamine dissolved in HBSS with a hand-held pipette at a concentration approximately equal to the purified protein EC_50_.

We also performed colocalization experiments to validate the correct expression of compartment-targeted iKetSnFRs. This approach used a red fluorescent probe (e.g., DsRed2: excitation 561 nm, emission 587 nm; mCherry: excitation 587 nm, emission 610 nm; mPlum: excitation 590 nm, emission 649 nm; mRuby: excitation 558 nm, emission 605 nm) that was specifically targeted by tagging with organelle targeting sequences and co-transfecting mammalian cells with both the probe and the compartment-specific iKetSnFR. To assess co-localization, Neuro2a cells were transfected, with the addition of 0.5 μg of red fluorescent protein (FP) complementary DNA (cDNA) to the transfection mixture. We had previously demonstrated proper localization of our ER-targeted constructs using the plasmid DsRed2-ER-5 (Addgene #55836; we also used mCherry-ER-3, Addgene #55041) in HeLa cells.

For PM-targeted constructs, images showing intense peripheral fluorescence with minimal internal signal in HeLa cells were sufficient to confirm correct localization. For nucleus-targeted constructs, clear labeling of the large nuclei of Neuro2a cells by iKetSnFR with exclusion from the soma indicated robust nuclear targeting. For cytoplasm-targeted constructs, exclusion from the nucleus together with iKetSnFR expression in soma and dendrites confirmed proper localization.

Among the new iKetSnFR constructs, we used the following markers for colabeling: mCherry-Peroxisomes-2 (AddGene #54520) for peroxisomes, mCherry-Mito-7 (AddGene #55102) for mitochondria, mCherry-Golgi-2 (AddGene #55052) for Golgi, mCherry-hLC3B pcDNA3.1 (AddGene #40827) for autophagosomes, and mCherry-Actin-C-18 (AddGene #54967) for F-actin (Key Resources Table).

### Ketamine metabolism and time-dependent detection of ketamine from the plasma of mouse blood

#### Plasma collection:

Whole blood was collected into EDTA tubes blood samples (~100 μL) from 4 mice per dose (10, 50, or 100 mg/kg IP or IN) were collected from the mouse tail vein at predetermined time points: 0 min, 15 min, 30 min, 60 min, 90 min, 3 h, and 6 h. Samples were centrifuged at 1,800 × g for 12 min at 4°C. The plasma supernatant was transferred to low-bind polypropylene tubes, flash-frozen on dry ice, and stored at −80°C until analysis. All procedures complied with institutional biosafety guidelines for handling animal plasma. No solvents were introduced prior to plasma separation.

#### Protein precipitation and small-molecule extraction:

Frozen plasma was thawed on ice and vortexed briefly. For each sample, 50 μL plasma was transferred to a 1.5-mL low-bind tube. Methanol (MeOH; LC–MS grade) was added at a 1:6 (v/v) plasma: MeOH ratio (i.e., 300 μL MeOH per 50 μL plasma). Samples were vortexed for 30 s and chilled at −20°C for 10 min to enhance protein precipitation. Tubes were centrifuged at 16,000 × g for 10 min at 4°C, and the clarified supernatant was transferred to a fresh tube, avoiding the pellet.

#### Concentration and reconstitution:

Supernatants were evaporated to dryness under vacuum (SpeedVac) at ≤ 4°C overnight. Residues were reconstituted in 50 μL MeOH (or 1:1 MeOH:H_2_O), vortexed for 60 s, and clarified by spin-filtration (0.2–0.45 μm). The final extract was transferred to LC vials with low-volume glass inserts and held at 4–8°C in the autosampler; all injections were completed within 24 h.

#### Mass spectrometry:

An Agilent 1260 liquid chromatography (LC) system coupled to a Thermo LCQ Deca mass spectrometer (MS) was used for LC–MS analysis, employing positive ion electrospray ionization (ESI). The ESI source was operated at a spray voltage of 5 kV, with a sheath gas flow of 80 units, an auxiliary gas flow of 20 units, and a capillary temperature of 250 °C. Chromatographic separation was achieved using an Agilent Zorbax SB-C18 column (4.6 mm × 150 mm, 3.5 μm particle size). The mobile phases consisted of water with 0.1% formic acid (A) and acetonitrile with 0.1% formic acid (B). The gradient was programmed from 5% to 95% B over 12 minutes, held at 95% B for 2 minutes, returned to 5% B in 1 minute, and equilibrated at 5% B for 5 minutes. The flow rate was maintained at 1.0 mL/min. Following UV detection, the LC effluent was split (1:5), directing 0.2 mL/min to the ESI source and 0.8 mL/min to waste. Data acquisition and processing were performed using Thermo Xcalibur software (version 2.0 SR2). Ketamine and its metabolites were primarily detected as protonated ions ([M+H]^+^) at m/z 238.1 (ketamine), 224.1 (norketamine), 240.1 (hydroxynorketamine), and 222.1 (dehydronorketamine). Due to the presence of a chlorine atom, ketamine and its fragment ions exhibit a characteristic isotopic pattern consisting of two peaks separated by 2 m/z units with an approximate 3:1 intensity ratio (^35^Cl:^37^Cl). This isotopic signature, together with LC retention time, was used to distinguish ketamine-related compounds from endogenous species.

### Pharmacokinetic modeling of ketamine following intraperitoneal and intranasal administration

Plasma ketamine concentration–time profiles were approximated using a one-compartment extravascular model [C(t) = A(e^−^ − e^−^)], based on published pharmacokinetic parameters^20,38^. This model assumes ketamine absorption into and elimination from a single, well-mixed compartment following non-intravenous administration. For i.p. administration, rapid absorption (k ≈ 0.35 min^−1^) and elimination (ln(2)/15 = 0.0462 min^−1^; ~15 min half-life) yielded a predicted peak concentration at ~7 min. For IN administration, slower absorption rates (k ≈ 0.12 min^−1^) were used to model delayed peak concentrations (~17.5 min), while maintaining the same elimination rate^98^. These simulations provide schematic representations of ketamine pharmacokinetics, as actual profiles vary with biological and experimental factors, including sex, strain, formulation, and sampling compartment. Moreover, IN pharmacokinetics are influenced by factors including delivery efficiency, mucosal absorption variability, and formulation.

### Mouse behavior recordings

All mice were maintained at the University of Pennsylvania, Perelman School of Medicine, John Morgan animal facility with a normal dark/light cycle and controlled room temperature and humidity conditions and had free access to food and water. All animal handling was under guidelines set forth by the School of Medicine’s Institutional Animal Care and Use Committee (IACUC), approved protocol number: 807237. C57/BL6 males at an age range of 8 to 12 weeks were used for behavioral experiments. Animals were housed in cages with five in each cage with ample food and water. All behavioral assessments were conducted following saline (control) i.p. injection, 10 minutes after ketamine (10 mg/kg, IP), and again at 60 minutes post-injection.

#### OFT:

The open-field test (OFT) was performed in a 50 × 50 cm open-field arena encased by plexiglass. The walls were covered to prevent visualization of the external field. The arena contained a 25 × 25 cm central region. The chamber was wiped with 70% ethanol and fully dried between animals. At the beginning of each trial, mice were placed gently into the center of the arena, and video tracking was started using the ANY-maze system. Over a 10-min session, time spent in the center, total distance traveled, and the proportion (%) of the total distance traveled being in the center.

#### RRT:

Rotarod test (RRT) was performed using the AccuRotor EzRod system (Omnitech; OH, USA). The rotarod was set to accelerate from 1 to 45 rpm over 80 s for a total period of 180 s. Mice were gently placed on the rod, allowed to stabilize, and a trial was started. Each trial began with the start of the rotation and ended when a mouse falls from the rotating rod. The test was repeated for 5 trials, and the average rpm of the trials was analyzed. Rod was cleaned with 70% ethanol and dried with task wipes before each trial.

#### HPT:

Hot-plate test (HPT) was performed using a Hot/Cold Plate Apparatus (Maze Engineers; IL, USA). Mice were placed on a pre-warmed surface maintained at 55°C for a maximum of 30 s. During each trial, three nocifensive responses were monitored and scored, reflecting spinal and supraspinal processing: (1) hind-limb flick (spinal reflex), (2) hind-limb licking or grasping (supraspinal/affective response), and (3) vertical jumping (escape behavior; supraspinal).

### EEG recording under ketamine

We utilized 6-pin head-mounts for electroencephalography (EEG) recording (Mouser; TX, United States). Electrodes were implanted epidurally over the left hemisphere to capture cortical local field potentials, and mice were allowed to recover for one week before recording. Signals were acquired using an 8202SL pre-amplifier connected to an 8206HR digital-to-analog converter (Pinnacle Technology Inc.; KS, United States). Data were digitized at 400 Hz and exported to MATLAB for analysis.

EEG traces were band-pass filtered between 0.5 and 50 Hz using a zero-phase Butterworth filter (filtfilt) and processed with a 60-Hz notch filter to remove electrical line noise. Spectral analysis was performed using the *pwelch* function (default parameters) with a non-uniform fast Fourier transform (NFFT = 512). In addition to power spectral density (PSD) estimation, density spectral arrays (DSAs; spectrograms) were generated using 5-s windows with 1-s steps. Error estimates were obtained by bootstrap resampling with replacement (1,000 iterations across windows) to derive 95% confidence intervals. Deviations from baseline were calculated by subtracting the mean baseline power spectrum from each post-drug spectral window.

### Two-photon iKetSnFR imaging in awake behaving animals

iKetSnFR was used for ketamine imaging of pyramidal neurons in the cortex. iKetSnFR expression was performed using intracranial AAV injections of neonates^99^. Pyramidal neurons were labeled using recombinant AAV1.hSyn.iSKetSnFR3.0 or AAV1.hSyn.iRKetSnFR2.0 (200 nL of AAV per mouse). Compartment-specific versions included AAV1.hSynapsin.IgK.iSKetSnFR3.0.PDGFR (for plasma membrane bound), AAV1.hSynapsin.NES.iSKetSnFR3.0 (for nuclear restriction). A glass micropipette (Drummond, 50001001X10) was pulled and beveled. A plunger was lightly oiled and inserted into the micropipette to pull the AAV mix. Subsequently, pups at postnatal day 1–2 were anesthetized by hypothermia (typically ~ 2–3 min on ice), and the micropipette was used (freehand) to penetrate the skin and skull and deliver ~200 nL of the virus mix. Medial prefrontal cortical (ACC) and primary visual cortex (V1) injection sites were determined using landmarks—skull suture lines and head veins—as reference points. Imaging was performed in 1 to 2-month-old mice, using both sexes, after at least 4 weeks of AAV expression. Mice were group-housed in temperature and humidity-controlled rooms on a 12-hour light/dark cycle after injections.

In preparation for imaging, mice underwent a surgical procedure to attach a head holder mount and create an imaging window for two-photon microscopy. In brief, mice were anesthetized with a mixture of 100% oxygen at 2 L min−1 and 1–4% isoflurane. A heating pad was used to maintain the animal’s body temperature at approximately 37°C. The mouse’s head was shaved, and its skull surface was exposed with a midline scalp incision. The periosteal tissue over the skull surface was removed without damaging the temporal and occipital muscles. A head holder consisting of two parallel metal bars was attached to the animal’s skull. In Cre-positive mice injected with AAV, <1% of mice were negative for iKetSnFR, suggesting off-target injection was a rare event. In positive mice, a small skull region (~2–3 mm in diameter) located over the interfrontal suture was removed, and a round glass coverslip (approximately the same size as the bone being removed) was affixed to the skull with Loctite 495 followed by dental acrylic cement. This window enabled imaging of ACC (+0.5–1.0 mm anterior of bregma and 0.1–0.3 mm lateral to midline). For V1, a cranial window was placed at 2.6 mm anterior of bregma and 2.6 mm lateral to midline. Upon recovering from surgical anesthesia, mice with head mounts were habituated daily (two sessions of 30 minutes with a 15-minute break) starting on postoperative day 1 in a custom-built body support to minimize potential stress effects of head-restraining and imaging. No obvious distress was observed in habituated animals during imaging experiments. Mice tolerated surgery and stress related to the perioperative period as indicated by a 0–10% drop in weight. Imaging experiments were performed on postoperative days 2–3 after window implantation.

On the day of imaging, awake mice were positioned in the custom head holder device under the two-photon microscope. *In vivo* two-photon imaging was performed with an Olympus DIY RS two-photon system (tuned to 910–920 nm) equipped with a Coherent Discovery NX laser. We minimized movement associated with image artifacts by head (secured metal head bars) and body (with a plastic sleeve) restraint on the imaging platform. In Fig. 4–6, mice were recorded using 0 mg/kg (saline), 0.1 mg/kg, 1 mg/kg, 5 mg/kg, 10 mg/kg, 50 mg/kg, and 100 mg/kg ketamine (Midwest veterinary supply) injected intraperitoneally. In Fig. 5, ketamine or saline (same volume) was delivered intranasally via a pipette application of a small volume (<10 μL). Pyramidal neurons in cortical regions were randomly chosen and recorded for 2-minute sessions under awake conditions and once again after ketamine application. Most experiments were performed using a ×20 Olympus objective (XLUMPLFLN; 1.00 NA, 2.0 mm working distance) immersed in aCSF, with ×2 digital zoom. In Fig. 5, ThorLabs TL10X-2P 10X super apochromat air objective was used. Images were acquired at a frame rate of 1–4 Hz (2-μs pixel dwell time). Image acquisition was performed using Olympus Fluoview software and analyzed post hoc using ImageJ software version 2.1.0.

During recordings, motion-related artifacts were typically less than 2 μm. Vertical movements were infrequent and minimized by two metal bars attached to the animal’s skull (described above) and a custom-built body support. All time-lapse images from each field of view were motion-corrected and referenced to a single template frame using cross-correlation image alignment (TurboReg plugin for ImageJ version 2.1.0). ROIs corresponding to visually identifiable somas (pyramidal cells) were selected manually from the field of view. Imaging planes were acquired from L2/3 corresponding to cells positioned ~50–350 μm from the pial surface, respectively.

All the pixels inside the ROI were averaged to obtain a fluorescence trace for each ROI. Background fluorescence was calculated as the average pixel value per frame from a region without iKetSnFR expression (blood vessel) and subtracted from the time-series fluorescence traces. The baseline (F_0_) of the fluorescence trace was estimated by the average of baseline (pre-ketamine) portions of the traces (~60 seconds). We did not smooth the raw fluorescence trace (raw traces are presented throughout the manuscript in each figure). The ΔF/F_0_ (%) was calculated as ΔF/F_0_ = (F_t_ − F_0_) / F_0_ × 100.

### Statistics

Summary data were presented as means ± s.e.m. No power analysis was used to determine sample sizes for Fig. 4–7. Data distribution was assumed to be normal, but this was not formally tested. The interventions for Figs. 4–7 were not blinded. In Fig. 5, group differences were evaluated using the Kruskal–Wallis test, with pairwise comparisons performed using Dunn’s multiple comparisons test. In Fig. 6, behavior data was analyzed using the Wilcoxon rank-sum test for pairwise comparisons between groups. All analyses were performed using custom scripts in Python or Prism 10. Data are presented as the mean ± standard error of the mean unless otherwise noted. Exact P values and common levels of significance (not significant (NS) P > 0.05; *P < 0.05; **P < 0.01; ***P < 0.001) are reported in figures, legends, and Supplementary Table 1. All statistical analyses were performed using GraphPad Prism.

## Supplementary Files

This is a list of supplementary files associated with this preprint. Click to download.

- SupplementaryInformationKalloletal2026.docx

## Figures and Tables

**Table:** Key Resources Table

Reagents or Resources	Source	Identifier	Additional information
R-(+)-Ketamine hydrochloride	Cayman Chemicals	Item No. 16519	RAAD Restricted Product Schedule III
S-(+)-Ketamine hydrochloride	Sigma-Aldrich	K1884	RAAD Restricted Product Schedule III
R-Norketamine hydrochloride	Biotechne-TOCR\S	5996	Ketamine metabolite
S-Norketamine	Biotechne-TOCR\S	6112	Ketamine metabolite
(2R,6R)-hydroxynorketamine hydrochloride	Sigma-Aldrich	SML1873	Ketamine metabolite
(2S,6S)-hydroxynorketamine hydrochloride	Sigma-Aldrich	SML1875	Ketamine metabolite
(R)-dehydronorketamine	Epichem	EPL-EG39	Ketamine metabolite
(S)-dehydronorketamine	Epichem	EPL-EG36	Ketamine metabolite
Nickel Sulfate [NiSO_4_]	Sigma-Aldrich	656895	For iKetSnFR purification
Dimethyl sulfoxide (DMSO)	Millipore Sigma	D8418	Solvent
Ethyl alcohol	Sigma-Aldrich	E7023	For DNA precipitation
**Critical Commercial Assays**			
*E. coli* BL21(DE3)	Agilent Technologies, Santa Clara, CA	200131	Chemically competent cells
*E. coli* TOP10	Thermo Fisher Scientific	C404050	Electrocompetent
NEB^®^ Stable Competent *E. coli* (High Efficiency)	NEB	C3040H	Chemically competent
*Dpn1*	NEB	R0176S	Restriction enzyme
*BamHI*	NEB	R0136S	Restriction enzyme
*Pst*I	NEB	R0140S	Restriction enzyme
*Eco*RI	NEB	R0101S	Restriction enzyme
*Hind*III	NEB	R0104S	Restriction enzyme
*Sal*I	NEB	R0138S	Restriction enzyme
*Not*I	NEB	R0189S	Restriction enzyme
*BglII*	NEB	R0144S	Restriction enzyme
*Agel*	NEB	R3552S	Restriction enzyme
*EcoRV*	NEB	R0195S	Restriction enzyme
*Quick CIP*	NEB	M0525S	Phosphatase enzyme
T4 DNA ligase	NEB	M0202S	DNA ligation
Ni-NTA Agarose	QIAGEN	ID. 30210	Protein purification
Phusion polymerase	NEB	M0530S	For PCR
dNTP mixture	NEB	N0447S	For PCR
Kanamycin sulfate	Millipore-Sigma	K4000	For bacterial culture
Ampicillin sodium salt	Sigma-Aldrich	A9518	For bacterial culture
Eagle’s Minimum Essential Medium (EMEM)	Thermo Fisher Scientific	50-238-2632	For tissue culture
Dulbecco’s modified Eagle’s medium (DMEM)	Thermo Fisher Scientific	12491015	For tissue culture
Fetal bovine serum	Thermo Fisher Scientific	26140	For tissue culture
DMEM/F12	Thermo Fisher Scientific	12634010	For tissue culture
HBSS	Thermo Fisher Scientific	24020117	For tissue culture
Penicillin/streptomycin	Thermo Fisher Scientific	15140122	For tissue culture
Lipofectamine 3000	Thermo Fisher Scientific	L3000001	For tissue culture
Trypsin	Thermo Fisher Scientific	20233	For tissue culture
DPBS	Thermo Fisher Scientific	14190144	For tissue culture
B27	Thermo Fisher Scientific	17504044	For tissue culture
Neurobasal medium	Thermo Fisher Scientific	10888022	For tissue culture
OptiMEM	Thermo Fisher Scientific	31985062	For tissue culture
RNase-free DNase	Thermo Fisher Scientific	89836	For cortical neuron culture
Papain PDS Kit	Worthington Biochem	LK003176	For cortical neuron culture
**Recombinant DNA**			
Plasmid: pEGFP-LC3 (human)	A gift from Toren Finkel	Addgene # 24920	Mammalian expression of LC3 fused to EGFP
Plasmid: mCherry-Lifeact-7	A gift from Michael Davidson	Addgene #54491	Localization: Actin, Excitation: 587, Emission: 610
Plasmid: mCherry-Peroxisomes-2	A gift from Michael Davidson	Addgene #54520	Localization: Peroxisomes, Excitation: 587, Emission: 610
Plasmid: mCherry-ER-3	A gift from Michael Davidson	Addgene #55041	Localization: Endoplasmic Reticulum, Excitation: 587, Emission: 610
Plasmid: mCherry-Mito-7	A gift from Michael Davidson	Addgene#55102	Localization: Mitochondria, Excitation: 587, Emission: 610
Plasmid: mCherry-Golgi-7	A gift from Michael Davidson	Addgene # 55052	Localization: Golgi Complex, Excitation: 587, Emission: 610
mRuby-Zyxin-6	A gift from Michael Davidson	Addgene #55887	Localization: Focal Adhesions, Excitation: 558, Emission: 605
mPlum-Fibrillarin-7	A gift from Michael Davidson	Addgene #55969	Localization: Nuclear - Nucleolus, Excitation: 590, Emission: 649
**Experimental Models: Cell Lines**			
Neuro2a Cells (Mouse)	ATCC	CCL-131	Neuronal and amoeboid stem cells
SH-SY5Y Cells (Human)	ATCC	CRL-2266	Neuroblastoma cell line
**Software and Algorithms**			
Origin9.0	OriginLab	Origin9.0	for interactive scientific graphing and data analysis
Excel	Microsoft	Microsoft	To graph, analyze and interpret data
PyMOL	Schrodinger, LLC	PyMOL Molecular Graphics System	Molecular visualization system
AutoDock Vina	Molecular Graphics Lab at The Scripps Research \nstitute.	https://vina.scripps.edu/	An open-source program for doing molecular docking
ChemDraw Professional 16.0	PerkinElmer	ChemDraw	ChemDraw is a molecule editor
ImageJ	NIH	ImageJ	ImageJ is a Java-based image processing program
FIJI	NIH	ImageJ	Fiji is an open-source image processing package based on ImageJ2.
CodonZ	NIH	CodonZ	Codon Optimization


## Data Availability

No custom code was created for this project.

## Reagent availability

All sensor constructs made here have been deposited at Addgene (**#XXX-XXX**).

## Data availability

All data needed to evaluate the conclusions and replicate the figures presented in this article are provided in the source data and associated data is available *via* figshare at**XXX**.Source data are provided with this paper.
